# Spatial Embedding and Wiring Cost Constrain the Functional Layout of the Cortical Network of Rodents and Primates

**DOI:** 10.1371/journal.pbio.1002512

**Published:** 2016-07-21

**Authors:** Szabolcs Horvát, Răzvan Gămănuț, Mária Ercsey-Ravasz, Loïc Magrou, Bianca Gămănuț, David C. Van Essen, Andreas Burkhalter, Kenneth Knoblauch, Zoltán Toroczkai, Henry Kennedy

**Affiliations:** 1 Univ Lyon, Université Claude Bernard Lyon 1, Inserm, Stem-cell and Brain Research Institute U1208, Bron, France; 2 Faculty of Physics, Babeş-Bolyai University, Cluj-Napoca, Romania; 3 Romanian Institute of Science and Technology, Cluj-Napoca, Romania; 4 Department of Anatomy and Neurobiology, Washington University School of Medicine, St. Louis, Missouri, United States of America; 5 Department of Physics, and the Interdisciplinary Center for Network Science and Applications, University of Notre Dame, Notre Dame, Indiana, United States of America; UT Southwestern Medical Center, UNITED STATES

## Abstract

Mammals show a wide range of brain sizes, reflecting adaptation to diverse habitats. Comparing interareal cortical networks across brains of different sizes and mammalian orders provides robust information on evolutionarily preserved features and species-specific processing modalities. However, these networks are spatially embedded, directed, and weighted, making comparisons challenging. Using tract tracing data from macaque and mouse, we show the existence of a general organizational principle based on an exponential distance rule (EDR) and cortical geometry, enabling network comparisons within the same model framework. These comparisons reveal the existence of network invariants between mouse and macaque, exemplified in graph motif profiles and connection similarity indices, but also significant differences, such as fractionally smaller and much weaker long-distance connections in the macaque than in mouse. The latter lends credence to the prediction that long-distance cortico-cortical connections could be very weak in the much-expanded human cortex, implying an increased susceptibility to disconnection syndromes such as Alzheimer disease and schizophrenia. Finally, our data from tracer experiments involving only gray matter connections in the primary visual areas of both species show that an EDR holds at local scales as well (within 1.5 mm), supporting the hypothesis that it is a universally valid property across all scales and, possibly, across the mammalian class.

## Abstract 2

The cortical network obeys common architectural principals in mouse and macaque. Differences include a relative decrease in long-range connections in the large brain of the macaque compared to the mouse.

## Author Summary

It was recently shown that the network of connections between different areas of the macaque cortex has strong structural specificity in terms of the strength of connections as a function of the distance between areas. This has led to a model of cortex connectivity that predicts many observed architectural features, including the existence of a strong core-periphery organization. When viewed across species, increases in brain size are accompanied by a relative decrease in connectivity, and thus an important question is whether there are architectural commonalities in the cortical networks within the mammalian branch. Here, based on tract tracing data from the folded macaque brain and the smooth mouse brain, we introduce a common model framework that allows network comparisons between species. We show that despite important differences in size, the cortices of both species share several network invariants, suggesting that the mammalian cortex exhibits universal architectural principals. This framework also captures differences between the two brains, including the fact that, unlike the macaque, the mouse core includes primary areas and that there is a relative decrease in the frequency of long-distance connections in the large macaque cortex compared to mouse. This approach allows network architectural extrapolations to the human cortex.

## Introduction

Understanding brain networks is arguably one of the major challenges of the 21st century [1]. The mammalian cortex is an extraordinary computational device, and analysis of its network properties with 10^7^–10^10^ neurons and 10^11^–10^15^ synaptic connections is still largely unresolved. In the brain, activity of a single neuron encodes relatively little information; instead, that is achieved via population coding, through spatially distributed temporal activity patterns of cell assemblies. This contrasts with packet-switching information technology (IT) networks, which encode information directly into the packets and the network merely ensures routing between any two nodes. Since the spatiotemporal activity of cell populations is strongly determined by their connectivity and physical layout, cortical network structure and its spatial embedding play a significant role in the brain’s processing algorithm, in sharp contrast with IT networks.

A purely bottom-up approach to deriving global brain function from local circuitry is currently intractable [2]. In contrast, a meso-scale approach is more feasible, focusing on the network of interactions between the elements of a mosaic of distinct areas representing the loci of function-specific computation (visual, auditory, somatosensory, motor, etc.). As the mammalian brain is shaped by evolution, morphological and areal network level inter-species comparisons will help identify those features that are conserved across species from those that are species-specific. This will lead to a better understanding of network structural properties and provide valuable clues to the evolution of brain function [3]. However, progress in this direction has been hindered due to the absence of (i) the necessary data to address the physical properties of the network between areas and (ii) adequate theoretical network comparison methods.

Published connectivity maps using consistent interareal tract tracing studies, first in the macaque [4] and more recently in the mouse [5,6], allow consideration of the network as a directed, spatially embedded and weighted graph (weights representing neuronal connection densities projecting between areas). The absence of full homology between the nodes (areas) and edges (projections) of the networks of the two species makes it difficult to determine commonalities and similarities between them. However, if generic, global organizational principles exist (constraining the adaptation and growth of cortical connections in similar ways), then we expect to see similarities at the statistical level between the network features in the two species.

Here we show that the cortical networks in the macaque and the mouse in fact do exhibit a common organizational principle despite their very different evolutionary trajectories and large differences in brain size. Supplemented by partial tract tracing data in the microcebus (the mouse lemur) we suggest that this principle and the associated network model is a universal determinant of the interareal network across mammals, allowing tentative predictions for the human brain.

Expansion of the cerebral cortex is accompanied by an increase in the proportion of white matter relative to brain size [7–10]. However, this increase is not rapid enough to maintain a constant neuronal connection density (defined as the fraction of neuron-to-neuron connections compared to all possible ones). Thus, an increase in brain size is expected to result in a reduction in the long-distance connectedness of cortical areas [11–14]. The reduction of the fraction of connections with cortical expansion and the minimization of the metabolic costs are important design features of the cortex [4,15–26]. One can hypothesize that this wire minimization constitutes a critical constraint for the optimal placement of areas in the cortex, serving to increase communication efficiency in larger brains [11,27–29], and is supported by recent evidence suggesting reduction of long-distance connectivity with increases in brain size [28].

Recent retrograde tract tracing data in macaque [30] provides supporting evidence precisely of such a wiring constraint, in the form of an exponential decay of the wiring probability *p*(*d*) with projection distance *d*: *p*(*d*)~*e*^−*λd*^, with a decay length (~1/*λ*) that is short relative to hemispheric dimensions (in the macaque *λ* ≅ 0.19 mm^−1^, corresponding to a decay length of $\frac{1}{\lambda }≅5.2\;\text{mm}$). A simple way to think of the decay length $\frac{1}{\lambda }$ is that every increase by $\frac{1}{\lambda }$ in projection length leads to a decrease in the number of projections by a factor of $\frac{1}{e}≅0.37$ (i.e., 37%). Note that using the base of the natural logarithm is convenient, as in this case $\frac{1}{\lambda }$ is equal to the average projection length, providing a simple, intuitive interpretation. We refer to this decay property of connection density with distance as the Exponential Distance Rule (EDR). Retrograde labeling using fluorescent tracers (see Materials and Methods section) is an accurate labeling method that reveals all incoming connections *j*→*i* to an injected (target) area *i* by labeling the cell bodies of the neurons in source area *j* whose axons make connections in area *i*. Importantly, there is no transneuronal labeling, so the retrograde labeling method used yields only one-step incoming connections to the injected nodes of the network.

Note that the EDR is purely a property of the distribution of the physical lengths of individual axons, without regard to any network topological structure. The EDR states that there are many fewer long-range axons than short ones and quantifies this: the number of axons of length *d* that we find in the cortex is proportional to *e*^−*λd*^. In general, to experimentally establish the EDR, we do not need to work with brain areas as nodes of a network; we only need to be able to count neurons and measure the corresponding axon lengths. In this sense, the EDR is a more basic and general property than the description of cortical connectivity as a network at some coarse-grained (e.g., mesoscale) level. Once the level of description is defined (e.g., areal), the network properties are, however, consequences of the distribution of the axonal lengths connecting the vertices. Since connectomes are embedded in physical space, the EDR property effectively constrains the topological structures that connectomes can form across different levels, ranging from the single neuron to the areal level [31].

In addition to the discovery of the EDR in the macaque, the consistency and completeness of this tract tracing data [32] has led to a deeper insight into the interareal network properties of the macaque cortex [30,33]: it revealed a much denser (*ρ* = 0.66) interareal cortical graph than previously reported (network density is defined as $\rho \;=\;\frac{M}{N\left(N-1\right)}$, where *N* is the number of areas and *M* is the number of connected ordered area pairs, see glossary). High density graphs have low specificity at the binary level (areas connected or not), so that what distinguishes one area from another is the particular combination of areas it is connected to, combined with the weights of the connections, i.e., their connectivity profile or fingerprint [33–36]. Because the range of weights spans many orders of magnitude (five in the macaque), the specificity of individual connectivity profiles is actually very high [5,30,37].

## Results

**Fig 1:**
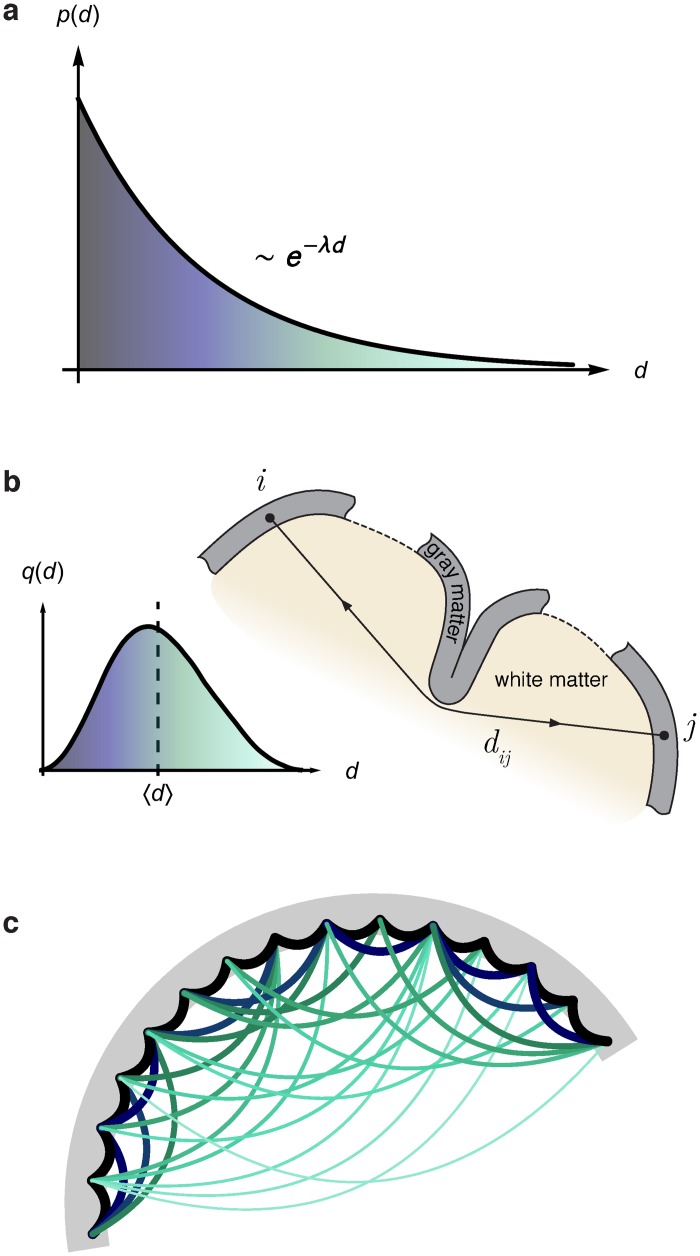
Schematic of EDR-based model of the cortex. **(a)** The exponential distance rule (EDR) expresses the empirical observation that the probability of axons of length *d* decay exponentially [4] with a decay rate *λ*. **(b)** Interareal distances *d*_*ij*_ are measured between the barycenters of the cortical areas *i* and *j* along the shortest paths through the white matter, avoiding the sulci and subcortical obstacles. The interareal distances follow a unimodal (Gaussian-like) distribution *q*(*d*) (i.e., *q*(*d*)Δ*d* gives the fraction of interareal distances with lengths between *d* and *d +* Δ*d*), as there are more area pairs separated at medium distances than at long or short distances, an observation valid for both smooth and folded brains (see Fig 7C). **(c)** The EDR network model (with the algorithm described in the text) generates strong connections (large bandwidth) between physically neighboring areas and exponentially decreasing strengths between areas that are increasingly far apart [4,40].

**Fig 2:**
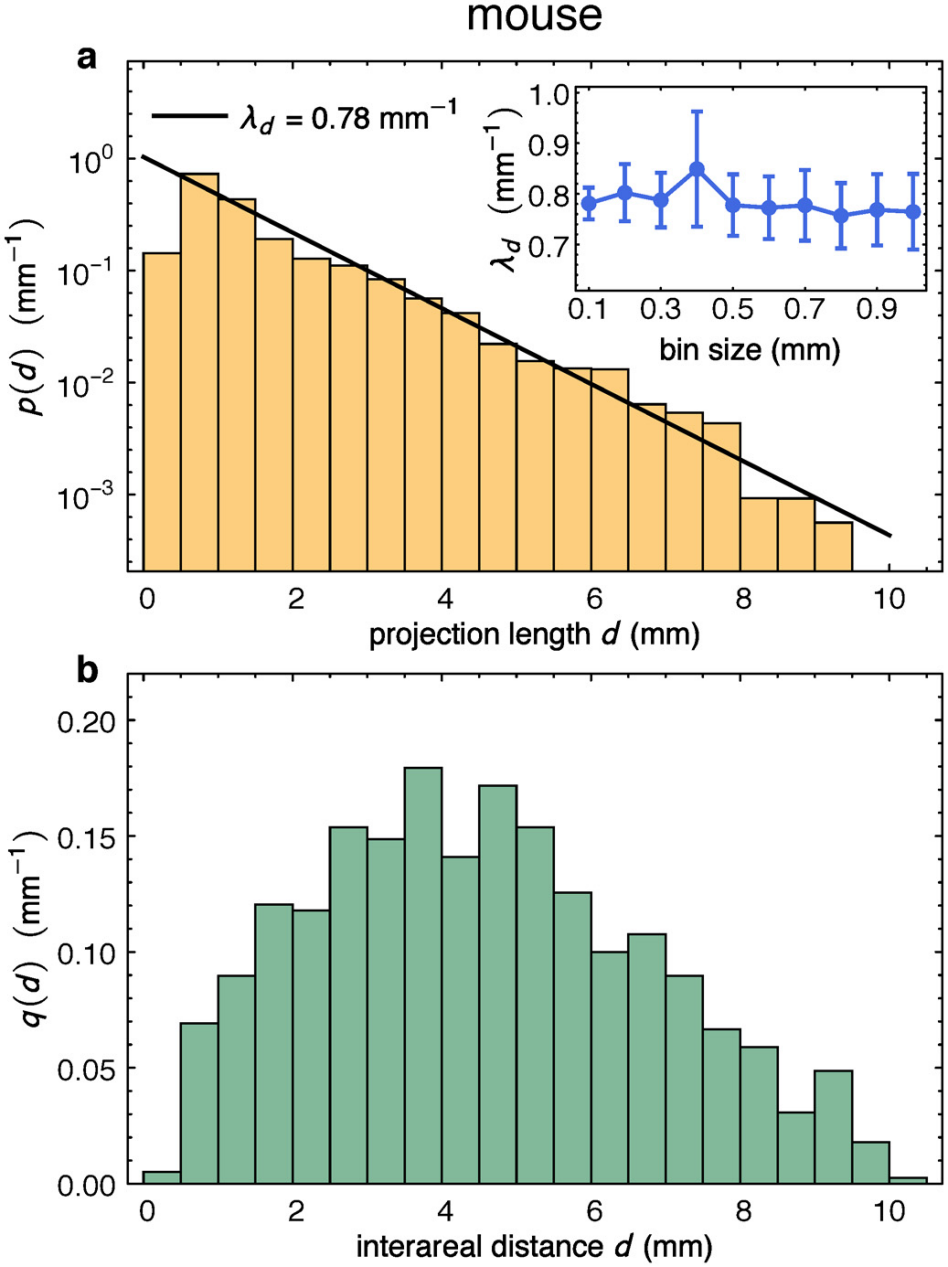
EDR parameters in mouse cortex. **(a)** Probability density function *p*(*d*) of direct neuron counts (about 2 million labeled neurons) versus distance from the injection site, generated using 13 retrograde tracer injections, see Materials and Methods. Inset shows the small variability of the fits for $\lambda_{\text{exp}}^{\text{mus}}$ as a function of bin size. The decay rate, $\lambda_{\text{exp}}^{\text{mus}}$, is determined by an exponential fit as $\lambda_{\text{exp}}^{\text{mus}}=\;0.78\;{\text{mm}}^{-1}$, with a 95% confidence interval of (0.72, 0.83) mm^−1^ (see inset). **(b)** Interareal distance distribution *q*(*d*) in the mouse on the flattened cortex, see Materials and Methods. First, the distance matrix *D*^mus^ was generated from flattened cortex measurements, then *q*(*d*) computed from these values. Accordingly, the edge weights (i.e., the FLN values) in the mouse are described by a log-normal-like distribution. Bin size in both plots is 0.5 mm.

**Fig 3:**
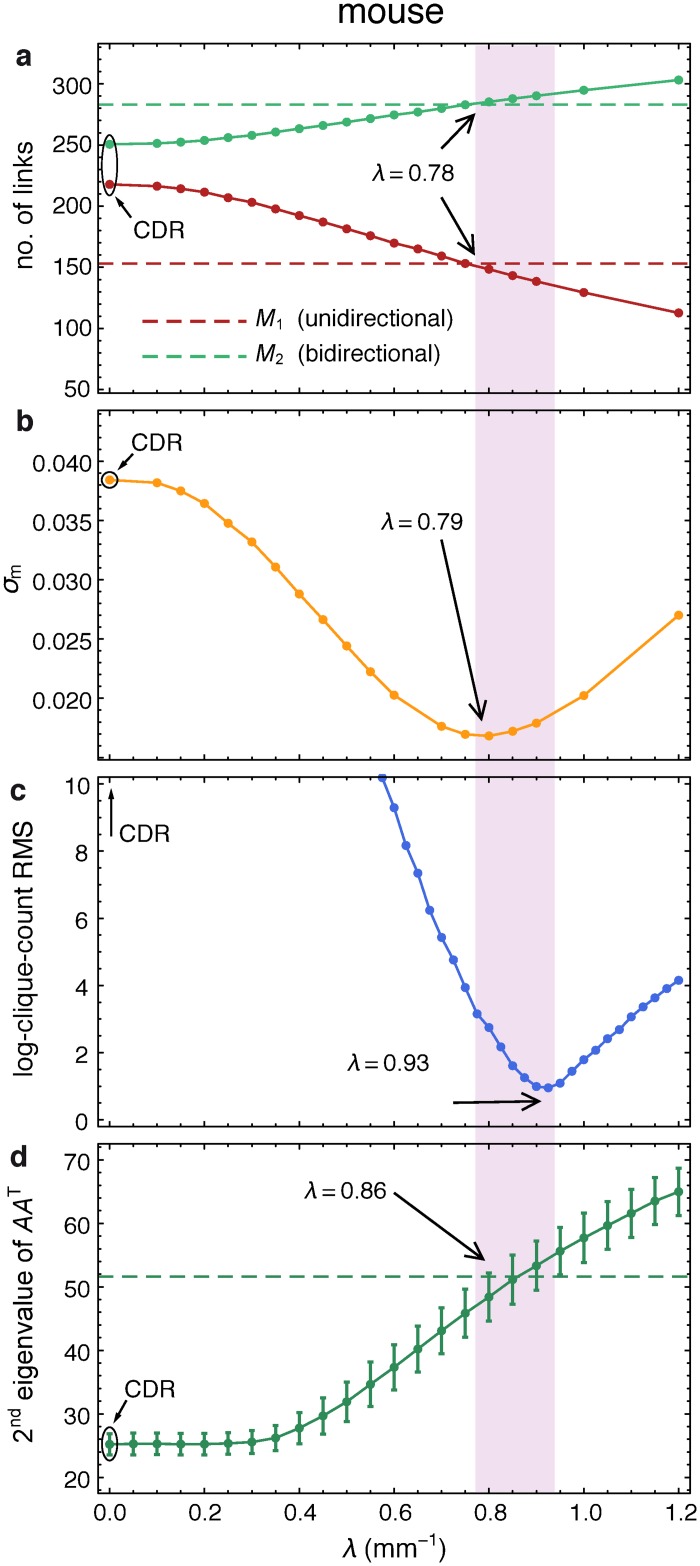
Consistency of fit by the EDR mouse model. **(a–d)** Determining *λ* by matching graph properties between the model and experimental data; data points show the average of 1,000 model-generated networks; vertical purple band: range of best fit for *λ*. Dashed lines in **a** and **d** indicate experimental data; **(a)** *M*_1_ and *M*_2_ represent counts of uni- and bidirectional links, respectively; **(b)** Root mean square (RMS) of log-ratios of 3-motif counts; **(c)** Root mean square of log-ratios of clique counts. **(d)** Second eigenvalue of *AA*^*T*^, where *A* is the adjacency matrix (using the co-occurrence matrix *AA*^*T*^, as it is a symmetric matrix and therefore has real eigenvalues).

We first give a schematic description of EDR-based network models (Fig 1) before developing a formal methodology for comparing EDR model graphs with experimentally obtained graphs, thereby allowing a quantification of the predictive power of the EDR network model for a given brain. This sets the stage for empirical measurements in the mouse brain, which are required for the construction of a mouse EDR model (Fig 2) and to examine how well the mouse EDR graphs fit with selected local and global mouse network properties obtained from empirical data (Fig 3). We next identify the core-periphery organization in the mouse network and show that it is well captured by the mouse EDR model. The following section is dedicated to a comparison of the capacity of the mouse and macaque EDR models in predicting empirically measured motif distributions (Figs 5 and 6) [38]. Analysis of network motif distribution is a recognized method of capturing the functional features of a network. The motifs analyses suggest the existence of common architectural features in the networks of both species; the following section analyses these structural commonalities by investigating the connection similarity index profiles between all node-pairs as a function of their spatial separation. However, in order to be able to perform comparisons involving distances in brains of very different sizes, we first introduce a common spatial template by an appropriate dimensional rescaling of the two brains. This allows us to show that, effectively, there is a common distribution of similarity indices as a function of adimensional separation in both brains (Figs 7 and 8). The finding that similarity changes across the cortex are only relatively consistent in the two species naturally leads us in the following section to consider the differences in cardinal features governing functional layout and to relate these differences to species characteristic properties of the cortex such as size and cortical folding (Fig 9). We conclude with a Discussion (Fig 10) in which we hypothesize that the EDR is a universal property across scales, i.e., valid also locally (through the gray matter), not just globally (through the white matter), and across the mammalian branch. As preliminary evidence supporting this hypothesis, we present results of tracer experiments (Fig 11) involving local connections only within the gray matter in three species—mouse, macaque, and microcebus—and quote results from other experiments in the rat. We conclude with mathematical arguments that further support the universal character of the EDR and speculate on the importance of these findings for understanding the human brain.

### EDR-Based Network Model of the Cortex

To what extent does the EDR, as a connectivity constraint, determine the properties of the interareal network? To address this issue, one needs (i) a family of EDR-based network models and (ii) a method of comparison between the model-generated networks and the experimental data network. The exponential decay rule ~*e*^−*λd*^ in the macaque was obtained from collating all the labeled neurons (over 6.4 million) following tract tracing experiments in different areas and constructing an interareal distance matrix, the latter estimated as the distances between the area barycenters through the white matter (WM), along the shortest paths. Here axonal *p*(*d*) should be interpreted as an average property (see Fig 1A), the probability that an axonal bundle projects to a distance *d*, independently of the specific functional nature of the areas. At this level of description, the strength of the connection between areas, expressed as the fraction of labeled neurons (FLN), depends uniquely on their geometrical separation. Thus, the network is viewed as a spatial, directed, and weighted graph dependent on the matrix *D* = {*d*_*ij*_} of interareal distances *d*_*ij*_. We emphasize here that the EDR arises from the estimated probability distribution of axon lengths. Although the strength-distance relation is consistent with the EDR, the probability distribution of axons lengths provides a more compelling demonstration of the property and leads naturally to the parametric EDR model described below. The probability density function, *q*(*d*), of the distances in the matrix *D* is typically a unimodal distribution (Fig 1B), which, when combined with the exponential decay *p*(*d*), leads to a log-normal distribution of edge weights, confirmed by the empirical FLN data [4,39].

The EDR distribution with the corresponding distance matrix *D* in a given brain naturally defines a parametric family of random graphs, called EDR random graphs (Fig 1C), parameterized by the decay rate *λ*. For these model graphs we make the choice *p*(*d*) = *λe*^−*λd*^, where now *λ* is the (only) model parameter. To distinguish the decay rate parameters in these models from the experimentally measured ones, we denote the latter as *λ*_exp_, e.g., for macaque $\lambda_{\text{exp}}^{\text{mac}}\;=\;0.19\;\text{m}{\text{m}}^{-1}$. We also employ, as a null model, the constant distance rule (CDR) family of random graphs, where there is no dependence of connection probability on distance, corresponding to the *λ*→0 limit, i.e., to the choice *p*(*d*) = const.

The EDR family of random graphs is defined via a simple algorithm [4] in the spirit of the Maximum Entropy Principle, i.e., it is based only on the given information (*p*(*d*) and *D*), while all else is uniformly random. The algorithm proceeds as follows: First, we randomly draw a connection length *d* from the distribution *p*(*d*). Second, we choose uniformly at random an area pair whose separation distance in the matrix *D* falls in the same distance bin as *d*, according to some binning criterion (bin sizes used in this study were typically 5 mm for the macaque and 0.4 mm for the mouse) and finally, insert a randomly oriented connection between them. Multiple connections between the same area pair in the same direction generate the weights for the directed edges with a log-normal distribution. These steps are then iterated until the graph density in the model reaches the observed value in the experimental network.

### Network Fitting and Comparison

We denote the data network obtained from the experiments by *G*_exp_ (e.g., for the macaque we use $G_{\text{exp}}^{\text{mac}}$, and for the mouse $G_{\text{exp}}^{\text{mac}}$). Our goal is to compare the properties of the EDR model networks with the properties of *G*_exp_. Since the model networks are only based on distance-dependent connection probabilities, one cannot expect perfect agreement (edge-by-edge) with the biological connectivity graph *G*_exp_, however, if the distance rule is a strong determinant of the interareal network, the model graphs should be statistically similar to *G*_exp_. The comparisons are performed via parameter matching of network properties [4]: for a given network property *P*, the interareal distance matrix *D* and parameter *λ* is used to generate a large ensemble of EDR graphs $G_{\text{EDR}}\left(\lambda \right)$. By varying *λ* we determine the value *λ*_*P*_ via minimizing the deviation |*P*(*G*_exp_) − 〈*P*(*λ*)〉|, with the average 〈∙〉 taken over at least 10^3^ EDR graph realizations from $G_{\text{EDR}}\left(\lambda \right)$. Thus the model parameter is determined so that the average of *P* in the model is as close as possible with the value of *P* observed in the data network. We then compare the fitted value *λ*_*P*_ with *λ*_exp_, the decay rate obtained directly from the experiments. If the two are close, then the EDR is a strong determinant for the measure *P* of the cortical network. Thus, the extent a particular measure in the EDR model and in the data network agree, i.e., |*P*(*G*_exp_) − 〈*P*(*λ*_*P*_)〉| with respect to the same comparison with the CDR model, i.e., with |*P*(*G*_exp_) − 〈*P*(*λ* = 0)〉|, expresses the degree to which the EDR influences that particular measure in the cortical network. This analysis is repeated with several local and global network measures. The more measures for which there is an agreement between *λ*_*P*_ and *λ*_exp_, the stronger the effect of the EDR in shaping the interareal network. This method also has the added advantage of identifying those network properties that are not well described by the EDR, and thus, based on the nature of these measures, providing us with clues for additional network mechanisms.

In the macaque, the EDR model predicts very well many local, global and weighted network properties of the interareal network (see [4] for details), and thus it is a strong determinant for the large-scale network organization of the macaque cortex. It also captures its pronounced core-periphery organization (i.e., a densely connected set of areas—core, with feedback and feedforward links to/from a more loosely connected set of peripheral areas), with the core strongly dominated by associative areas [4,40].

The EDR network model of cortical connectivity represents a radical departure from previous, purely topological models of cortical networks, which do not take into account their physical, i.e., weighted and spatially embedded nature, and this has now been well documented in the recent literature [41,42]. The spatial clustering and geometrical positioning of the nodes in the EDR model in the macaque is observed to strongly echo the functional layout of the cortex as revealed by numerous physiological and anatomical studies [36,43].

### The EDR in the Mouse and the Associated Network Model

To determine whether a similar description is valid for the mouse (*Mus musculus*) cortex, we first conducted retrograde tracer experiments in the mouse neocortex (S2 and S3 Figs), in order to determine the projection length distribution *p*(*d*), which, indeed, shows a clear exponential decay (Fig 2A). The decay rate, $\lambda_{\text{exp}}^{\text{mus}}$, was determined from an exponential fit as $\;\lambda_{\text{exp}}^{\text{mus}}\;=\;0.78\;{\text{mm}}^{-1}$, with a 95% confidence interval of (0.72, 0.83) (see Fig 2A, and inset). This exponential decay is to be compared with the same distribution for the macaque from Fig 2B in Ref [4] (see Table 1 for the *λ* parameter estimates). The distance matrix *D*^mus^ was determined from flattened cortex measurements. The corresponding distance distribution *q*(*d*) is unimodal, as shown in Fig 2B, which is to be compared with the same distribution for the macaque from Fig 2C in reference [4] (the consequences of the differences in these distributions are discussed in more detail in the section “Functional Layout in Terms of Spatial Clustering of Cortical Areas”).We then applied the network analysis described in the section above to the largest available edge-complete graph (the status of connectivity between all pairs of nodes is known) of 33 areas in one hemisphere of the mouse neocortex [5,6], denoted by $G_{\text{exp}}^{\text{mus}}$ from here on (S1 Fig). This mouse dataset contains 719 directed pathways and has an interareal network density of *ρ*^mus^ = 0.68, similar to that reported in the macaque.

**Table 1:** Summary of EDR parameters in the mouse and macaque.

	mouse	macaque
*λ*_exp_(mm^−1^) (white matter)	0.78	0.188
$\lambda_{\text{exp}}^{\text{local}}$ (mm^−1^) (gray matter)	4.61	4.46
〈*d*〉 (mm)	4.54	26.35
*γ* = *λ*〈*d*〉	3.54	5.0

Fig 3 shows the proximity of $\lambda_{P}^{\text{mus}}$ obtained using the parameter matching method to the decay rate $\lambda_{\text{exp}}^{\text{mus}}$ for several network measures including the number of area pairs connected uni- (*M*_1_) or bidirectionally (*M*_2_), 3-motifs, clique distributions and the second largest eigenvalue of the symmetrized form *AA*^*T*^ of the adjacency matrix *A*. These measures have been selected in part because they probe graph properties from local to global scales, and are of varying complexity. Additionally, these measures (see glossary for definitions), and in particular the deviations from their values in random graphs carry functional significance: unidirectionally connected areas depict an asymmetric role in information processing (driver versus driven nodes), the 3-motifs have extensively been studied as building blocks of functional organization in complex networks [38], cliques identify maximally connected network regions usually representing activity-specific strongly correlated communities or clusters, and the second largest eigenvalue is related to the rate of spreading processes (e.g., epidemics or information) on the network [4].

The different comparisons and fits based on these measures are highly consistent and indicate *λ* to be in the range 0.78–0.93 mm^−1^ (purple vertical band in Fig 3A–3D). The broader range in mouse of $\left|\lambda_{P}-\lambda_{\text{exp}}^{\text{mus}}\right|$ compared to macaque might be due in part to the fact that the mouse connectivity matrix was generated by anterograde tracing [5,6]. Other statistical network properties, such as degree distributions, are likewise well captured by the EDR network model with *λ* = 0.78 mm^−1^, (see S4 Fig).

### Core-Periphery Structure in the Mouse Cortex

**Fig 4:**
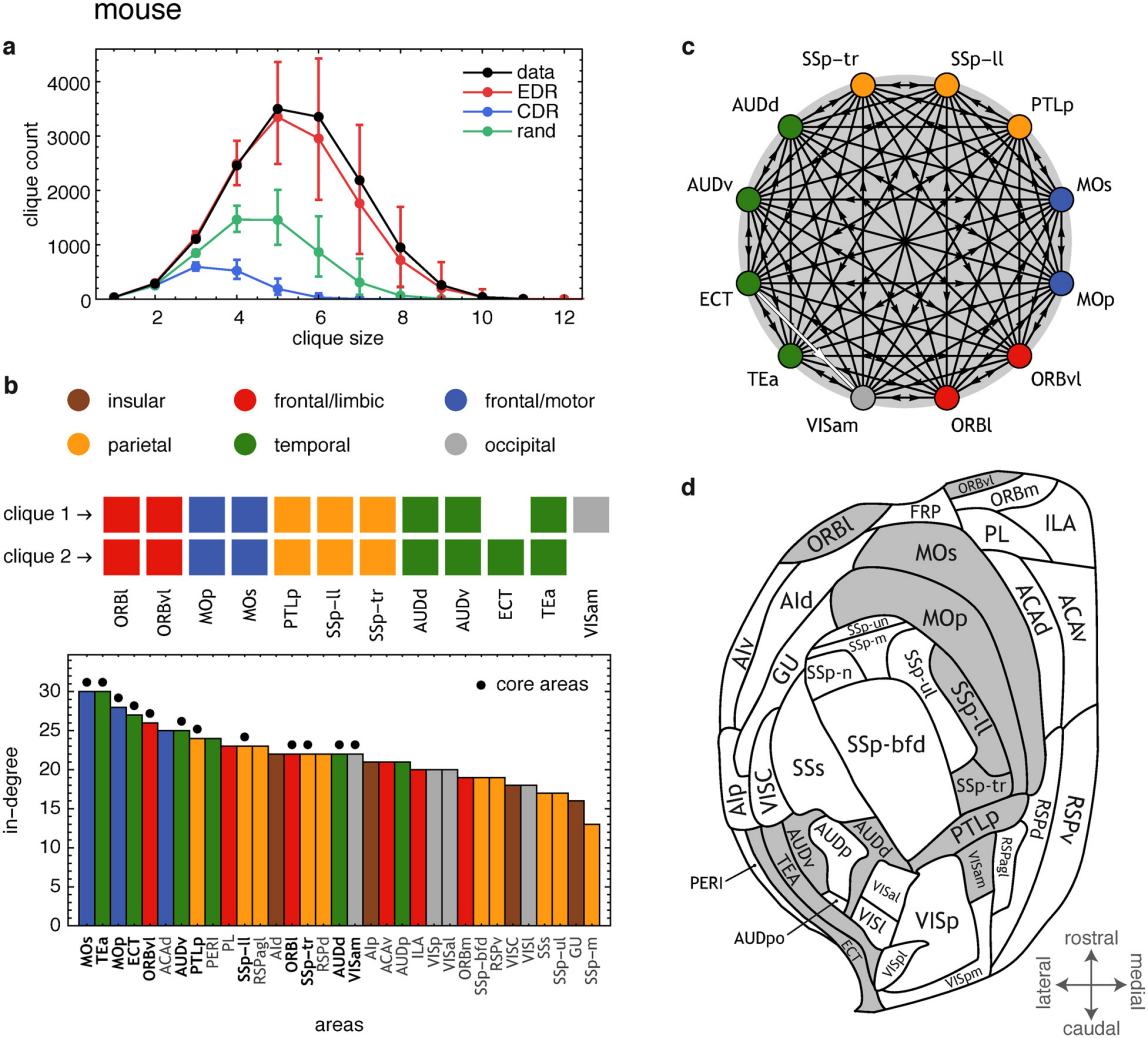
Clique distribution and core-periphery structure in mouse. **(a)** Clique distribution compared between empirical **data**, **EDR** model (*λ* = 0.93 mm^−1^, best fit from Fig 3C), **CDR** model, and a **rand**omized network with the same degree sequence as the data. **(b) top**, mouse network core composed of two cliques of size 11, shown as two rows of squares, each square representing an area that is part of the clique. Three primary areas are present (MOp, SSp-ll, SSp-tr); **bottom**, in-degrees of mouse cortical areas. Dots mark core areas, largely centered on the highest in-degree areas, consistent with the macaque [30]. **(c)** The white arrow (ECT → VISam) shows the single missing link between the 12 members of the core. **(d)** Flat map of mouse cortex; gray color represents network core members.

*A clique* (see glossary in S1 Text) is a complete subgraph of a network, i.e., it carries the maximum number of possible edges between its nodes. In dense graphs (thus with many cliques) the size (number of nodes) distribution of the cliques provides insight into the network’s heterogeneity [4]. The largest cliques in dense graphs can be used to define the cortical network core [4,40]. As in macaque [4,40], the clique distribution analysis in the mouse (Fig 4A) reveals a distinct core-periphery structure. The mouse connectome, $G_{\text{exp}}^{\text{mus}}$, includes a dense core of 12 nodes organized into the two largest cliques each of size 11, plus a periphery of 21 nodes. There are a total of *M*_*cc*_ = 131 links within the core, *M*_*cp*_ = 190 links from the core to the periphery, *M*_*pc*_ = 170 from periphery to core, and *M*_*pp*_ = 228 links within the periphery. Densities for the mouse are the following: core 99% (versus 92% in macaque), periphery 54% (versus 49%) and the links between the core and periphery, 71% (versus 54%). The likelihood of a core having 12 nodes in a random graph on 33 nodes with the same density *ρ* = 0.681 as in $G_{\text{exp}}^{\text{mus}}$ is vanishingly small: $\left(\begin{array}{c} 33\\ 12 \end{array}\right)\left(\begin{array}{c} 132\\ 1 \end{array}\right)p^{131}{\left(1-p\right)}^{1}\;=\;2.07\times 10^{-12}$ (versus 10^−17^ in the macaque). To see how well the EDR model reproduces the clique distribution, we define a scalar deviation measure *σ*_cl_(*λ*) between the clique-size distributions in the data and the EDR model as the root mean square (RMS) of the clique-count log-ratios. The best agreement between the two distributions is achieved at $\lambda_{\text{cl}}^{\text{mus}}\;=\;0.93\;{\text{mm}}^{-1}$ (Fig 3C) and the clique distributions in the model and data are rather close at this value (Fig 4A).

Anatomically, the cortical core in the mouse shows significant differences with that previously reported in macaque, the most striking being that the mouse core includes portions of primary somatosensory cortex (SSp-ll and SSp-tr) and primary motor cortex (MOp) (Fig 4B). While additional injections may well expand the core membership in macaque, primary areas in the macaque core are extremely unlikely, given the rarity of connections linking primary areas [30]. This contrasts with the mouse where the inter-primary area subgraph has a density of over 80% [5,6]. In agreement with the presence of primary areas in the mouse core, the two-dimensional map of the flattened cortex (Fig 4D) shows that the mouse cortical core might be spatially more widespread across brain regions compared to that of the macaque, where the core appears concentrated in frontal and parietal areas [4]. Note that in both mouse and macaque, the core areas have overall, higher in-degrees than non-core areas (Fig 4B for the mouse). The wider spatial spread of the mouse compared to the macaque core may reflect the relative expansion in primates of higher-level association cortex with respect to the primary areas [3]. These differences in the cortical core of the mouse and macaque need to be considered in light of the proposal that in primates at least, the core is related to cognitive architectures such as the global workspace, thought to be involved in consciousness [40,44].

### Motifs Comparisons between Mouse and Macaque

**Fig 5:**
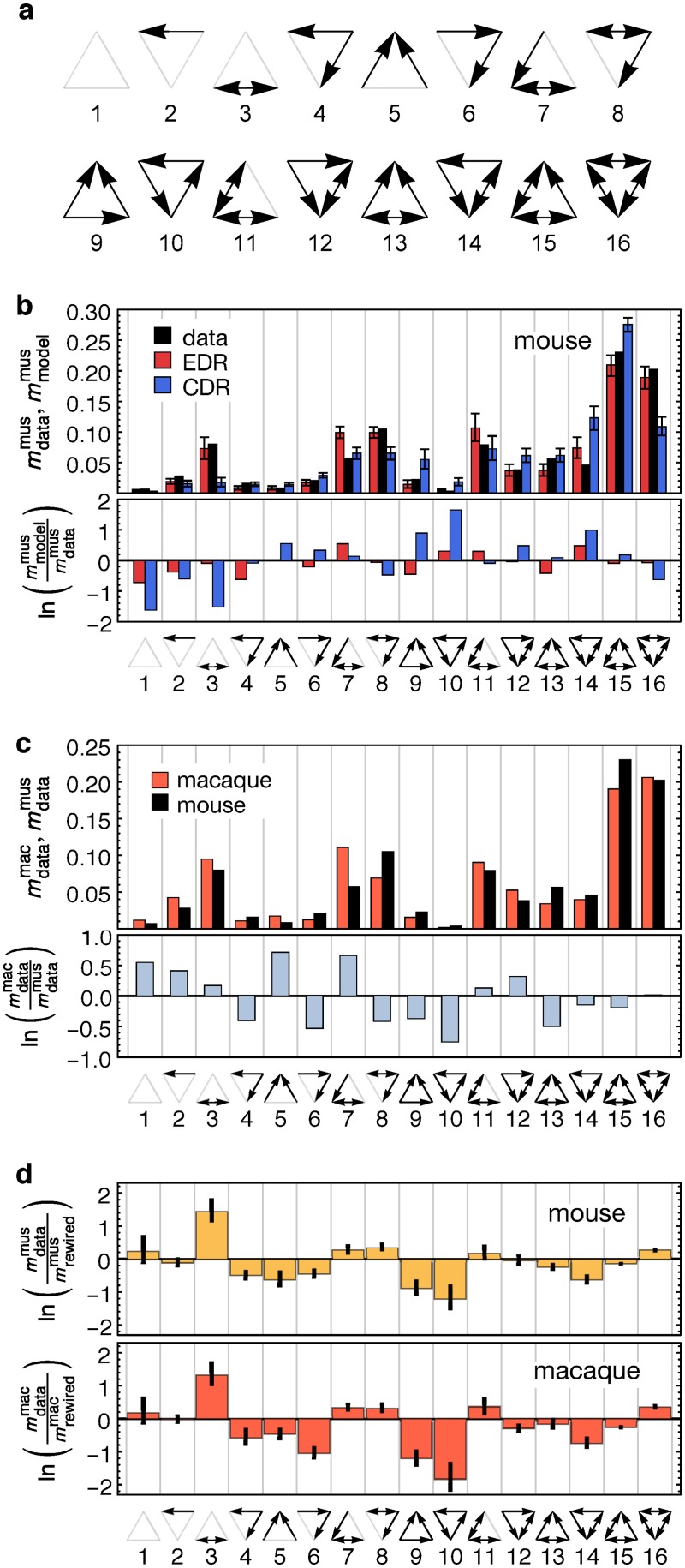
Three node motif profiles of mouse and macaque connectomes. **(a)** Full complement of three node motifs, i.e. all possible connectivity patterns found for three nodes **(b)** Mouse motif distribution, EDR (*λ* = 0.78 mm^−1^), CDR models and data comparison. Note that the EDR captures much better the motif frequencies than does the CDR. The average of 1,000 model networks is shown; error bars represent 95% confidence intervals. **(c)** Motif distributions of macaque and mouse, comparison. **(d)** Motif distributions of mouse and macaque connectomes compared to a null model (logarithmic residuals) obtained from random rewiring of edges while preserving the in- and out-degree sequence. The average of 1,000 rewired networks is shown; the error bars show 95% confidence intervals.

Network motifs refer to the different possible connectivity patterns of a small, fixed number of nodes. For example, in the 33-node mouse cortical network there are $\left(\begin{array}{c} 33\\ 3 \end{array}\right)\;=\;5456$ triplets of nodes, each of which has one of the 16 connectivity patterns shown in Fig 5A. Three-node motifs have been proposed as the building blocks of network circuits and their pattern of variation in frequency to reflect functional properties of the networks [38]. For instance, motif 10 (oriented 3-cycle) is significantly under-represented in the cortex, while motif 3 is significantly *over*-represented (lone, bidirectional link) when compared to a random network, in both species (see Fig 5D).

As in macaque [4], the mouse EDR predicts the observed motif frequency distributions in this species significantly better than does the CDR (Fig 5B). Despite the marked quantitative differences in motif distributions between mouse and macaque (Fig 5C), there could, however, be qualitative similarities. Testing this requires comparing the observed motif profiles to that of a randomized null model [38] consisting of an ensemble of random networks having the same degree sequence as the data. Graphs were uniformly sampled from this ensemble by repeatedly rewiring edges [45].

**Fig 6:**
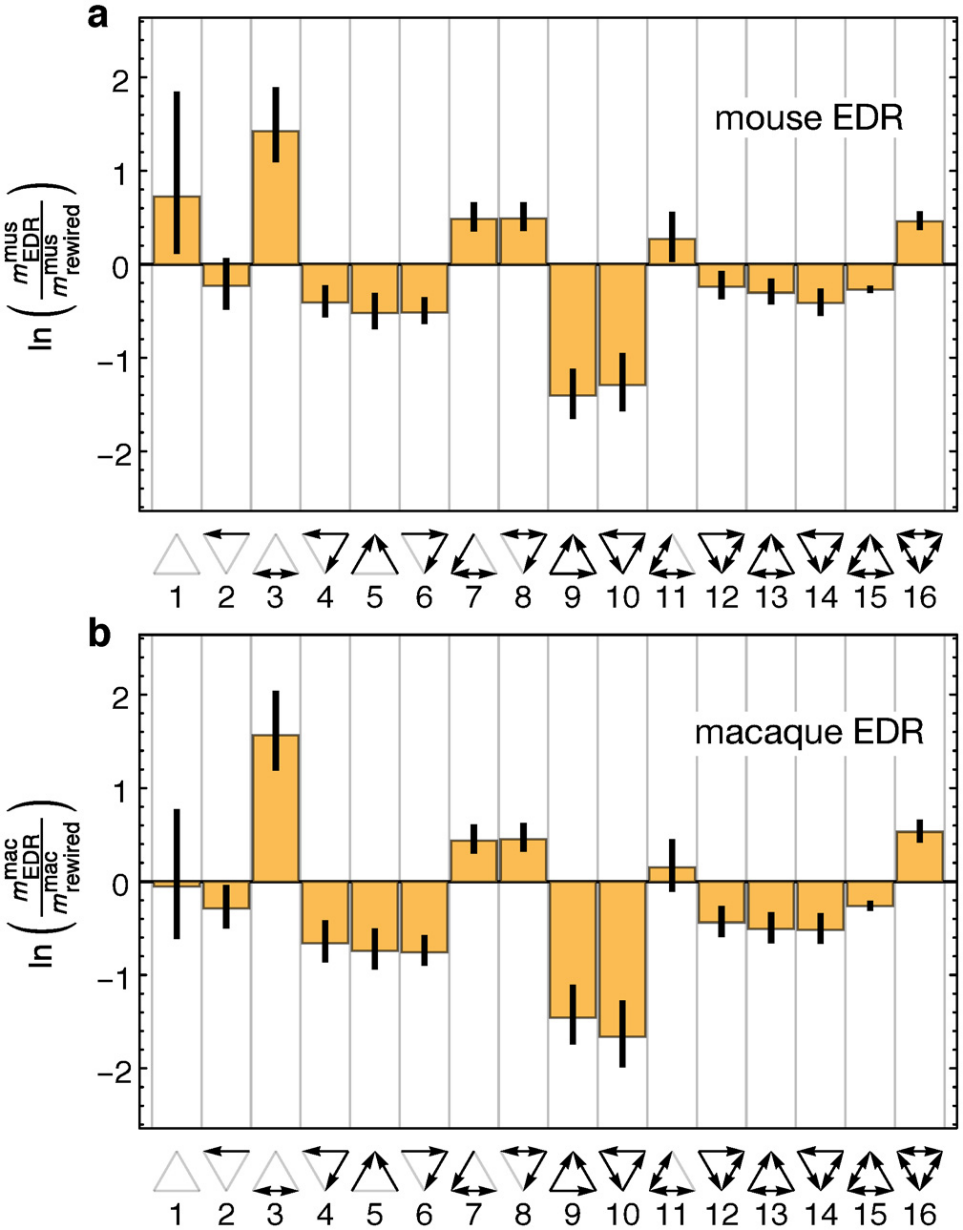
Macaque and mouse Motif distributions of EDR networks compared to a null model. The null model used for motif comparison is obtained from the network to which it is to be compared by a random rewiring of its connections in such a way as to preserve the in- and out-degrees. **(a)** Comparing motifs between the EDR model based on the macaque distance matrix and decay rate *λ* = 0.19 mm^−1^ and the associated null model. **(b)** Same as in (a) but for the EDR model based on the mouse distance matrix, *λ* = 0.78 mm^−1^. This figure is analogous to Fig 5D but uses EDR model generated networks for comparison with null models instead of the empirical ones.

Fig 5D shows how the motif counts of the empirical connectomes differ from such a randomized null model. As similar patterns are observed in both species, these findings suggest that they are part of the same class of large-scale networks with similar architectural and functional constraints. Repeating this analysis for networks generated by the EDR model (see Fig 6) we find a remarkable similarity in the motif profiles not just between the networks of the real data network and the EDR model but also between the model networks of the two species (Fig 6). This confirms the existence of a common network architectural invariant in these two species. This is unexpected, insofar as the decay rates and the distance matrices are very different between the two cortices. Since the motif profiles are binary measures, these findings indicate graph structural similarity between the two brains. In order to test whether there are significant similarities in the large-scale connectomes of the two species beyond the constraints imposed by the EDR, we used the EDR model as a null model [46]. S5 Fig shows that motif counts continue to look similar between the mouse and macaque, although their similarity is now less pronounced. To further probe the wiring similarity between the mouse and macaque connectomes, we next study the connectivity similarity profile measure.

### Comparing Mouse and Macaque Cortical Networks within a Common Spatial Template

Elsewhere we have demonstrated that a quantitative measure of the similarity of the connectivity profiles of target cortical areas decreases in a regular fashion with increasing distance between them, i.e., the closer two target areas are, the more their source areas overlap [4,33]. We have also shown that changes in similarity reflect the functional layout of the cortex [33], and thus it is natural to compare the behavior of this measure between the mouse and macaque.

**Fig 7:**
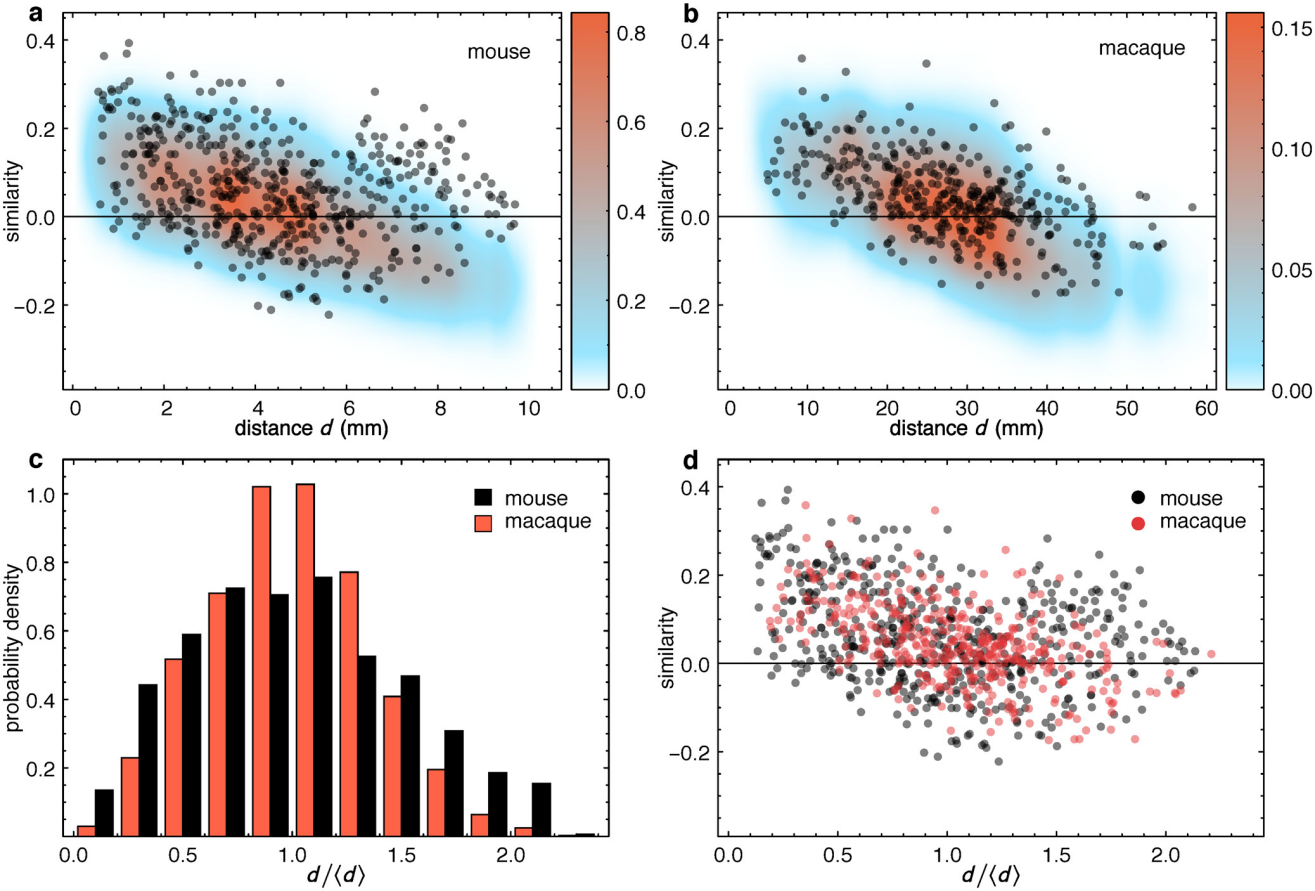
Connection similarity profiles on a common spatial template in mouse and macaque. **(a)** Mouse connection similarity indices for all area pairs (33 × 32/2 = 528 points) versus the separation distance between the area pairs. **(b)** Same as (a), but for the macaque. Color range, smoothed probability density for similarity indices measured in 300 random networks generated by the EDR model for each species. Note the agreement between the connectome data points and the EDR distribution. **(c)** Distribution of distances *q*(*d*/〈*d*〉) from the distance matrices on the common, adimensional brain template. In mouse we divide all distances in the distance matrix by 〈*d*〉^mus^ = 4.54 mm, in macaque distances divided by 〈*d*〉^mac^ = 26.35 mm. Bins shifted for better visibility. Bin size, 0.2 (adimensional). **(d)** Mouse and macaque connection similarity indices, show significantly overlap in the adimensional brain template.

A similarity index [4] can be defined for both incoming (in-link similarity) and outgoing (out-link similarity) connections. In order to compare macaque and mouse similarity indices, we focus here on the incoming connections, as those are the ones fully specified for all the injected areas in the macaque dataset. Next, we analyze the similarity between the connectivity profiles, for all possible target area pairs. The in-link similarity index for any two (target) areas is a measure describing the extent to which both targets receive/or do not receive in-links from the same source areas, compared to a fully randomized state of the network (see Materials and Methods section for details). Fig 7 shows the distribution of in-link similarity indices as function of the distance between all area pairs in the mouse (Fig 7A) and the macaque (Fig 7B). In both species, in-link similarity decreases with increasing distance between the area pairs, i.e., areas that are further apart on the cortical sheet have increasingly dissimilar in-link connectivity profiles on average, while the opposite is true for areas that are closer to one another. The colored regions in both Fig 7A and 7B are the probability densities of in-link similarity indices generated by the corresponding EDR models, with red corresponding to higher, and blue to lower probabilities; in both cases the EDR model captures the average behavior rather well.

**Fig 8:**
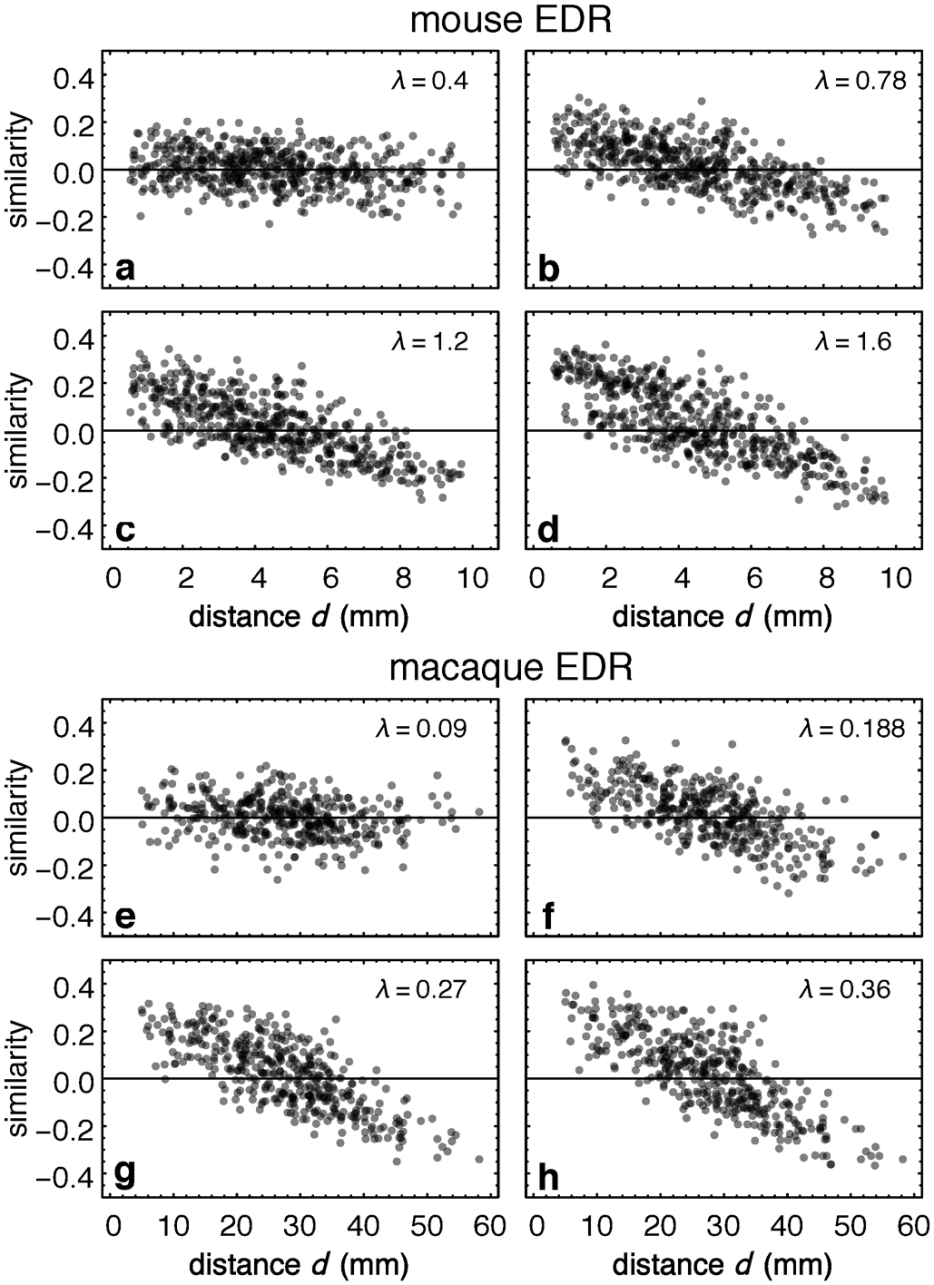
Mouse and macaque connection similarity indices in the EDR networks as function of the decay rate *λ*. **(a–d)** mouse, **(e–h)** for the macaque. Data points generated from a typical sample EDR model network for each species.

In order to compare distance-dependent quantities between brains of very different sizes, all distances are rescaled by the average interareal distance in each species (〈*d*〉^mus^ = 4.54 mm and 〈*d*〉^mac^ = 26.35 mm). Interestingly, as the largest distances are $d_{\text{max}}^{\text{mus}}\;=\;10.1\;\text{mm}$ and $d_{\text{max}}^{\text{mac}}\;=\;58.2\;\text{mm}$ respectively, this fits both brains onto the same adimensional template, as $\frac{d_{\text{max}}^{\text{mus}}}{d^{\text{mus}}}\;=\;2.22\;\text{mm}$ and $\frac{d_{\text{max}}^{\text{mac}}}{d^{\text{mac}}}\;=\;2.21\;\text{mm}$. Fig 7C shows the corresponding distribution of adimensional distances *q*(*d*/〈*d*〉). When plotting the in-link similarity indices against the rescaled distances (Fig 7D), we find a remarkable overlap between the clouds of points in the two species. This is rather surprising given the fact that they have very different decay rates *λ*. They also have rather different interareal distance matrices as the macaque cortex is folded, resulting in it having a more peaked distance distribution than the mouse (Fig 7C). Fig 8 shows the sensitivity of the in-link similarity indices using the EDR models in both the mouse (panels 8A–8D) and the macaque (8E–8H). For a given distance matrix, the point clouds are observed to rotate as a function of *λ* in both species, and hence there is no a priori reason for the overlap in Fig 7D. This overlap, however, is an indication of the existence of a network architectural invariant, present in both species, also reflected in the motif profiles discussed earlier. Further explanation for the significant, overall overlap between the similarity distributions for the two species is provided in the Discussion section.

### Functional Layout in Terms of Spatial Clustering of Cortical Areas

**Fig 9:**
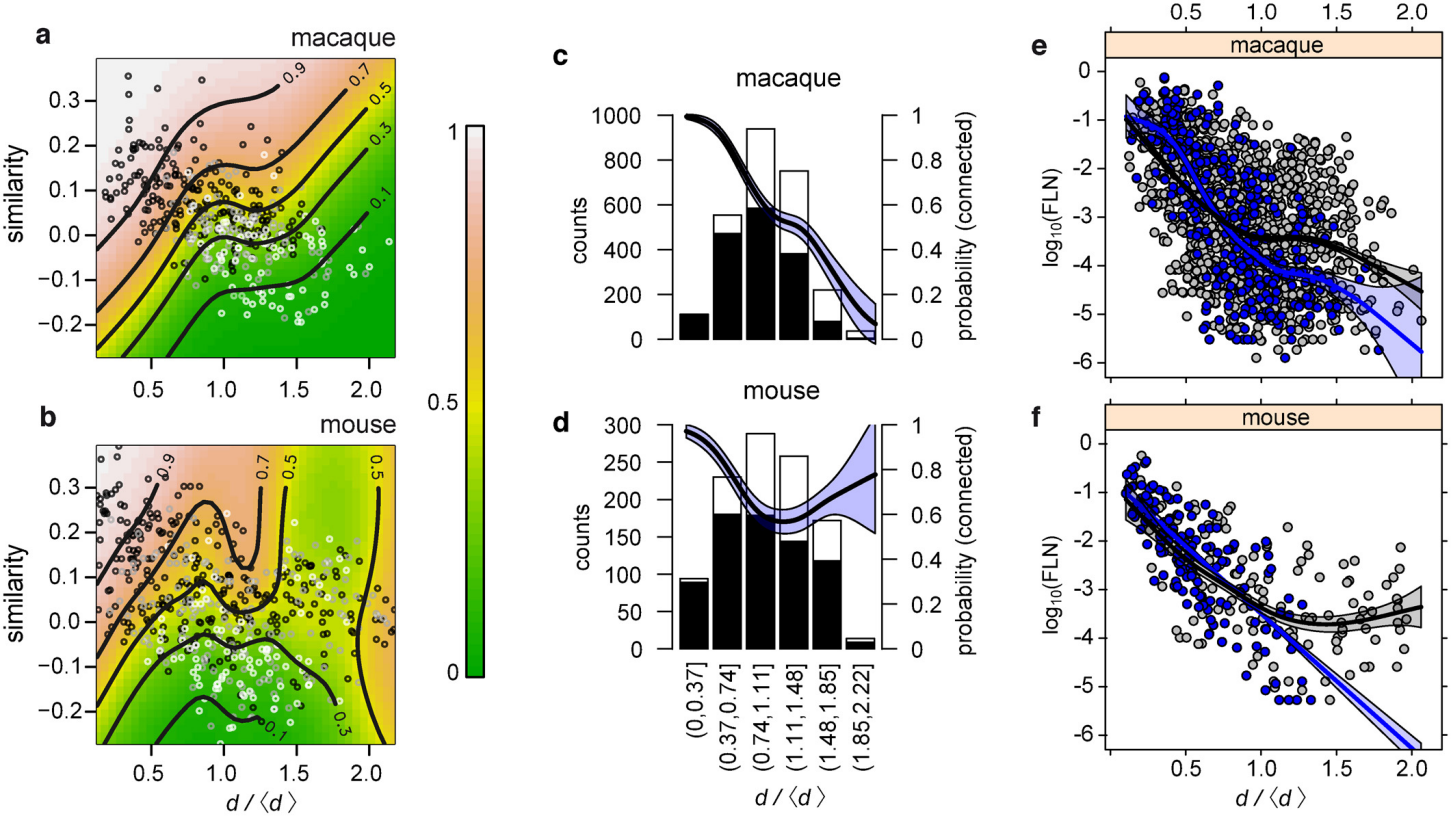
Mouse and macaque differences in functional layout. **(a,b)** Smooth estimates of the probability that an area pair is connected as a function of similarity and normalized distance. Circles (○) show connected (black) or unconnected (white) area pairs. Overlapping black and white circles appear as gray. Curves indicate contours of constant probability; color gradient shows probability values. **(a)** Positive contour slope for macaque indicates probability dependency on distance and similarity. **(b)** This relation breaks down for mouse at large distances. Macaque and mouse differences are significant, *χ*^2^(8.4) = 61.6, *p* = 3.5 × 10^−10^. **(c,d)** Histograms showing the number of connected and non-connected areas at given distance intervals from injected target areas for **(c)** macaque and **(d)** mouse. Black bars, connected source areas; white bars, nonconnected areas. Smooth curves indicate decay of connection probability with normalized distance in macaque and mouse; a Generalized Additive Model (GAM) with a binomial family and logit link was fitted for each species (solid lines), curves differ statistically *p* < 3.05 × 10^−12^ (see Materials and Methods). **(e,f)** FLN values as a function of normalized distance for connections running among canonical areas (blue) and all other connections (among association areas, or between an association area and a canonical area, gray), for the **(e)** macaque and **(f)** mouse connectomes. Regression lines are smooth estimates of the trends in the data obtained with additive model fits. The blue curves with standard error envelope are for the canonical areas and the black curves with standard error envelopes for the associative areas. The species differences between the fits are significant (*χ*^2^(5.17) = 22.97, *p* = 4.4 × 10^−4^).

With the help of the common adimensional template defined above we now discuss species-specific characteristics in our comparison of cortical networks. The EDR decay *p*(*d*) can simply be recast in terms of adimensional distances, by writing $\left(d\right)\sim e^{-\lambda d}\;=\;e^{-\gamma \;\frac{d}{d}}$, where *γ* = *λ*〈*d*〉 is the adimensional (or normalized) decay rate. Accordingly, *γ*^mus^ = 0.78 × 4.54 = 3.54 and *γ*^mac^ = 0.19 × 26.35 = 5, showing that on the common template, the mouse has a shallower connectivity decay than the macaque. The distribution of distances in the mouse is broader compared to the macaque (Fig 7C), which when coupled with the shallower connectivity decay contributes to the mouse cortex experiencing a less constraining effect of the EDR than does the macaque. This difference in the EDR between the two species explains some of the differences in the functional layout of the cortex in mouse and macaque. In Fig 9A and 9B we show the same similarity indices for all area pairs as before but also indicate which area pairs are connected (black circles) and which are not (white circles) and provide smooth estimates (colored regions) of connection probability as a function of similarity and adimensional distance. Comparing Fig 9A and 9B we see that in macaque, spatially clustered, presumably functionally related neighboring areas are heavily interconnected and share similar connectivity profiles, while more distant areas show weaker probability of connectivity and similarity index. This relationship between probability of connectivity, spatial separation and similarity is, however, weaker in the mouse.

In both species, connection probability changes as a function of distance. Fig 9C and 9D show how this relationship differs in the two species. Consistent with a steeper EDR in the macaque, neighboring areas exhibit 100% connectedness, and the probability of connections (density) decreases smoothly and consistently with distance to around 10% density at maximum distances [47]. This contrasts with the mouse, in which neighboring areas do not quite reach densities of 100% and widely separated areas have densities in the region of 50% to 80% (Fig 9C). Hence, these results show that compared to macaque, in the mouse, widely separated areas are more likely to be interconnected. These differences in the probability of being connected as a function of distance between the two species appear highly significant (smooth curves in Fig 9C and 9D).

Numerous studies point to the cost of long-distance connections as an inherent design challenge associated with differences in brain size [48]. One way to define total wire length is: Λ = Σ_*i*,*j*_*A*_*ij*_*D*_*ij*_, where *A* denotes the binary adjacency matrix and *D* is the interareal distance matrix. Yoked permutations of the rows and columns of the adjacency matrix reassign the distances to each pair of areas while maintaining the connectivity unchanged. As in macaque [4], the total wire length of the mouse inter-areal network is significantly shorter than a random permutation of the areas (S6 Fig). Simulated annealing methods [4] showed that optimization of area placement can lead to a 12% reduction in total wire length in the mouse, significantly higher than the 5% reduction obtained in macaque [4].

Next, we address the strength of connections with the expectation that long-range connection strengths (expressed as FLNs) would decrease in the larger brain. Due to the EDR, the FLN clearly decreases with distance. Distinguishing interareal association and canonical connections allows an improved understanding of the effect of distance on connection weight (for definition of associative and canonical connections see [3]) (Fig 9E and 9F). This suggests that the decline in FLN is steeper in canonical cortex compared to association cortex, so that the long-distance association cortex connections are one to two orders of magnitude stronger than the connections between canonical cortex areas with the same separation (see [3]). However, the results suggest that the decline in weight with distance is steeper in the macaque compared to the mouse. Together these findings show that compared to the larger macaque cortex, in the smaller mouse brain long-distance binary interareal connections are marginally more numerous. By contrast there is a highly significant increase in the weight of the long-distance connections in the mouse, and this species difference is more pronounced in the projections of association than in the canonical connections of the primary areas.

## Discussion

The present meso-scale network investigation of the neocortex, with appropriate network comparisons, provides detailed information on a common organizational principle that explains numerous network features in two widely separated species, with distinct evolutionary histories. Based on phylogenic considerations, and the fact that evolution is essentially a tinkerer [49], one expects to find evolutionarily preserved features embedded in these networks, i.e., architectural invariants. Evolutionarily preserved features, however, often are expected to manifest themselves as organizational principles tied to biophysical constraints.

**Fig 10:**
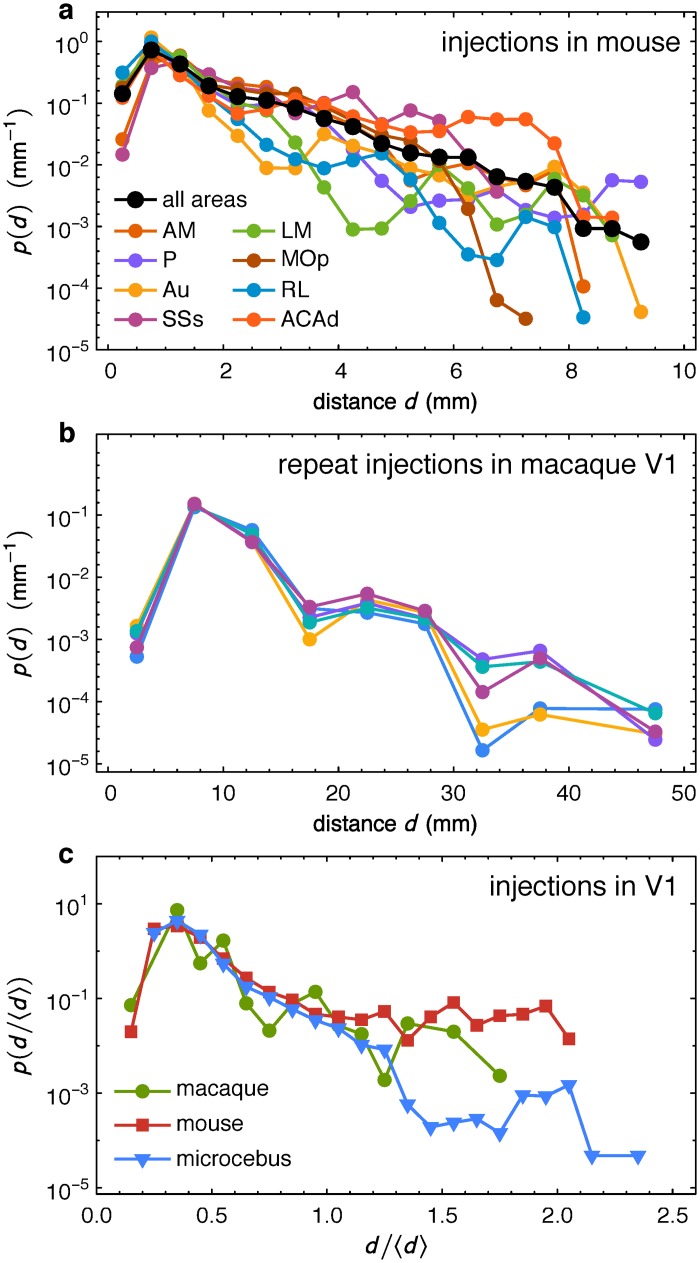
The EDR describes an average property. **(a)** Connectivity probability decays for eight different targets (colors) in the mouse. Black curve, overall distribution (averaged over all targets), same as in Fig 2A. **(b)** Connectivity probability decays in the macaque from V1 as target, in five different individuals, showing that fluctuations are consistent. This indicates that the variability around a perfect exponential decay for a specific target is not just noise. **(c)** Decays for V1 as target on the adimensional template brain in three species: macaque (green circles), mouse (red squares), and microcebus (blue triangles).

The success of the mammalian class includes adaptation to diverse habitats and lifestyles, which is in part attributed to the behavioral flexibility ensured by the neocortex [50]. The modulation of corticogenesis [51] has led to extant mammals exhibiting a five-orders of magnitude range of brain size [52], going from small-brained mammals that include miniaturization of ancestral forms to the expansion and additional arealization that characterize primates, especially humans. The present results suggest that the EDR plays a key role across the mammalian order to optimize the layout of the inter-areal cortical network allowing larger-brained animals to maintain communication efficiencies combined with increased neuron numbers. Our results indicate that the EDR and the associated network model provide a unifying framework to capture common network properties but also some of the differences across the mammalian branch and thus allow network comparisons between species. The EDR decay rate *λ* and cortical geometry (interareal distances) significantly impact on the structural heterogeneity of the cortical network with important consequences for the general functional layout and core-periphery structure, that we speculate, could be involved in higher cognitive processes [40]. The limitation of the EDR model stems from the fact that the EDR describes an overall, or average property. At this level, without additional determinants, it should not be used as a generative model of individual connections as we have emphasized elsewhere [4,40]. If we plot the decay of the probability of connections for several target areas, as shown in Fig 10A for the mouse, we find significant variability. The black line in Fig 10A, the average decay, is the same as that in Fig 2A. The fluctuations for a given target, however, are not noise, but rather they are part of a signal. This we illustrate in macaque: Fig 10B shows the consistency of fluctuations following repeat injections in area V1, in five different individuals. There are numerous factors that one might need to take into account to better understand this variability. For example one may need to consider the observed systematic variation in neuron numbers across the cortex [53,54], the anisotropy of axon outgrowth distributions [55] and possibly diverse developmental factors [56]. Overall, however, these considerations emphasize that the EDR network serves as a framework, upon which other details are imposed.

Note that in order to assess the ability of the EDR model (or any connectome model) to reproduce properties of empirical network data, it is crucial that the data is as edge-complete as possible, i.e., that the connectivity between any two nodes is known. Otherwise the lack of fit between model and data cannot be used to discard the model [57]. This holds for two reasons: (a) the EDR network model produces complete connectivity information between its nodes, it cannot generate “untested” connections, by default, and (b) many network measures can be sensitive to the absence or presence of an even a small fraction of connections in the network. It is also important to emphasize the roles of cortical geometry [58] and that of areal segmentation in shaping the network properties of the connectome. Since the connection probability depends on distance, network properties are influenced by the relative proximity of areas. In turn, the strength of connections between functionally defined areas correlate with the amount of signaling activity between them and therefore with their functional roles within the information processing hierarchy in the brain. Ad-hoc segmentations, however, will generate ad-hoc distance matrices for the EDR model, and accordingly, the model networks would no longer be interpretable from a functional circuitry point of view, and in this sense, it is important to use optimally defined functional parcellation of the cortex.

Our comparative analysis of motifs and connectivity similarity indices demonstrates the existence of network architectural invariants, which in turn imply that the EDR parameter *λ* and areal positioning (geometry) are not independent parameters: while both change during evolution, the changes are orchestrated in such a way as to ensure that certain network/circuitry properties are preserved. As argued in the introduction, the network, i.e., the graph connectivity (form) must play a significant role in the information processing algorithm itself (function), and thus these network invariants are a reflection of common processing dynamics in the cortex.

Our use of a normalized or adimensional distances facilitates comparisons across brains of different sizes. Fig 10C shows directly the fingerprint of such universal principles in neocortical organization: it shows the connection probability decay on the adimensional template brain from a common target area (area V1) in macaque (data from reference [30]), mouse as well as microcebus. At short to medium distances where the vast majority of neurons are located, decays are identical, but are observed to change in a species dependent fashion for the long-range connections. Microcebus belongs to a group that contains the smallest existing primates, with a brain under 2 cm in length. Although the microcebus data is only for V1, it remarkably fits to the same adimensional template, with a decay rate *λ* between that of mouse and macaque, suggesting that the quantitative differences that distinguish the species are due to both brain size, and primate-rodent differences.

**Fig 11:**
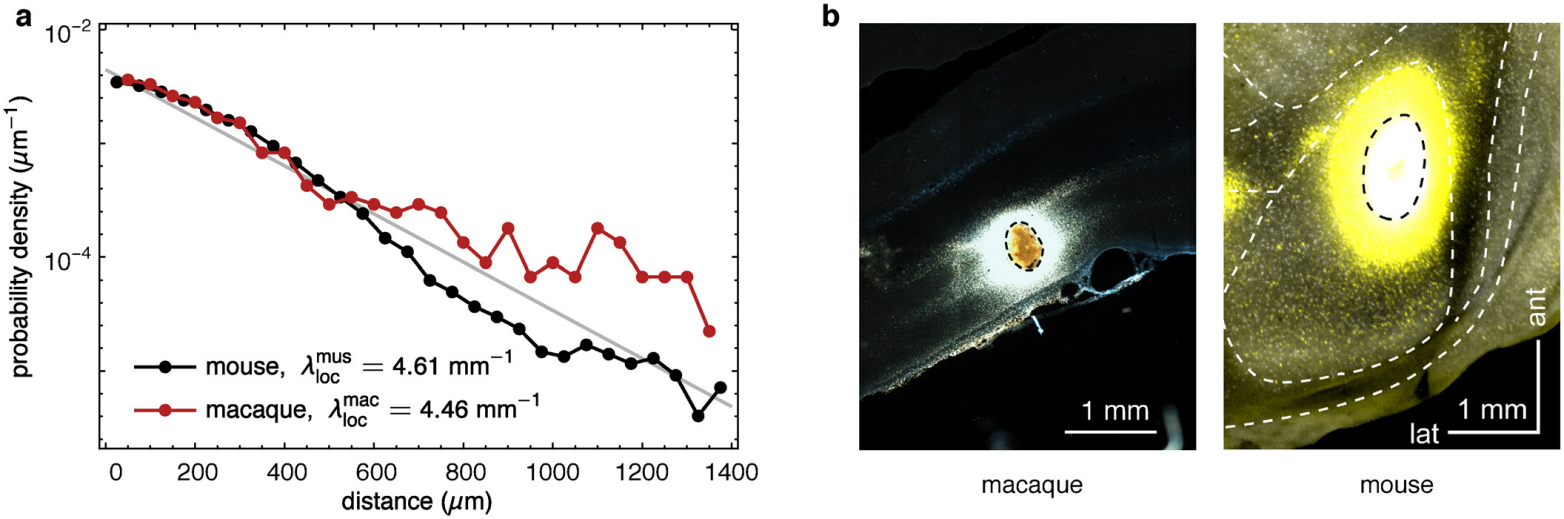
Decay rates of intrinsic labeling in mouse and macaque. Intrinsic retrograde labeling in macaque and mouse show a common, exponential decay of the connection probability with distance in the gray matter measured from the edge of the pickup zone. **(a)** Probability density function of neuron counts as a function of the distance from the edge of the pickup zone. The 95% confidence intervals of the decay rates are (4.58, 4.65) mm^−1^ for mouse and (4.14, 4.80) mm^−1^ for macaque. The mouse decay rate is estimated for the interval up to 900 μm using a generalized linear model with a Poisson family and log link. **(b)** Photographs of injection sites in area V1 in macaque (horizontal section) and mouse (tangential section). The black dashed line corresponds to the edge of the pickup zone in both panels.

The EDR could be the expression of the consequence of a universal information processing principle implemented in the cortex across several scales, specifically to include single neurons in the local circuit, which present over 80% of the cortical connectivity [31,39]. Hence, the two major ingredients for the EDR are found in the local circuitry, the log normal distribution of synaptic weights [59] and an exponential decay of connection distances as reported here. Further, the experimental evidence presented in Fig 11, shows that *p*(*d*) follows a nearly identical, exponential decay out to within 1.5 mm for both mouse and macaque, with $\lambda_{\text{exp}}^{\text{local}}≅4.54\pm 0.08\;{\text{mm}}^{-1}$. These are gray matter, non-myelinated connections, and are observed to have a very different decay rate than white matter connections. Thus, at least in area V1, the decay of connectivity with distance seems to behave in a very similar fashion in both mouse and macaque, and therefore surprisingly the decay rate in the gray matter does not appear to be related to brain size. Using the reported data in [59] for the rat visual cortex obtained from quadruple whole cell recordings, the local decay rate in the rat can be determined to be $\lambda_{\text{exp}}^{\text{local}}≅4.96\;{\text{mm}}^{-1}$, a value consistent with the one found above in mouse and macaque, above. Table 1 summarizes the EDR related parameters in the mouse and macaque, for both white matter and gray matter connections.

The universal character of the EDR is further supported by mathematical arguments. The exponential distribution (EDR) is memoryless (Markov property), i.e., in our case, the probability that an axon of some length grows by an additional amount is independent of its current length (within cutoff limits). In this way, it has the property that f(d+l) = f(d)f(l), where $f\left(l\right)\;=\;\int_{l}^{\infty }p\left(l^{\prime }\right)dl^{\prime }\;=\;e^{-\lambda l}$ is the probability of an axon growing to a length beyond l. The exponential distribution is the only continuous distribution with this property [60]; for all other distributions, growth depends on the current length, i.e. on past growth history. This also implies that the EDR is the maximum entropy probability distribution for axonal lengths with given expectation value (= 1/*λ*), see [61]. These properties are evolutionarily advantageous, conferring maximum adaptability during cortical expansion. Moreover, as more neurons are added, the probability distribution of the shortest connection among an arbitrary number of other connections also obeys an exponential distribution [62], making the EDR an invariant property *locally* as well, supported by the experimental data quoted above.

The present findings could have important consequences for understanding the human brain. The recognized limitations of current tractographic analysis of diffusion MRI data [63,64], means that direct observation of long-distance connections in the human brain is not presently feasible. Given the specificity of long-range cortico-cortical connectivity [47], this technical limitation has important consequences for understanding the human connectome, and we believe that comparative connectomics as developed in the present study will be a necessary step for determining universal principles of cortical connectivity. Hence, an in-depth understanding of the influence of changes in brain size will play an important role in better understanding the human brain. Since the EDR leads to a decrease in the strength of long-range connections in macaque compared to mouse, we may hypothesize that increase in brain size leads to increased reductions of weight in long-range projections for the whole mammalian branch. In the human brain the small number of fibers in such long distance connections will pose an acute problem for detection for some time. This could constitute an important limitation. For example, one could speculate that the low weight of human long-range connections may contribute to an increased susceptibility to disconnection syndromes, such as have been proposed for Alzheimer disease and schizophrenia [65–67].

## Materials and Methods

### Tract Tracing

Experiments were performed in male and female PV-Cre [68] (Jax: 008069), x Ai9 reporter mice (Jax: 007905), harboring the loxP-flanked STOP cassette, which prevented the transcription of the tdTomato protein driven by the chicken β-actin (CAG) promoter [69]. The crossing produced Cre-mediated recombination, which resulted in the expression of the red fluorescent protein in the subset of parvalbumin (PV)-positive GABAergic neurons. All experimental procedures were approved by the institutional Animal Care and Use Committee at Washington University and conformed to the National Institutes of Health guidelines.

Injections were made in *Microcebus murinus* in area 10 and area V1. Surgical and experimental procedures were in accordance with European requirements 2010/63/UE and approved by the ethics committee CELYNE (ref 00439.02).

### Tracer Injections

For tracer injections, mice were anesthetized with of a mixture of Ketamine (86 mg · kg^−1^) and Xylazine (13 mg · kg^−1^, i.p) and secured in a head holder. The body temperature was maintained at 37°C. Intracortical connections within the left hemisphere were retrogradely labeled by inserting a glass pipette (20 μm tip diameter) into the brain and injecting Diamidino Yellow (50 nl, 2% in H_2_O; EMS-Chemie, Gross-Umstadt, Germany) by pressure (Picospritzer, Parker-Hannafin). Injections were performed stereotaxically 0.35 mm below the pial surface, using a coordinate system whose origin was the intersection between the midline and a perpendicular line drawn from the anterior border of the transverse sinus at the posterior pole of the occipital cortex. The injections were made in the following areas: V1, RL, AL, LM, P, RSD, ACAd, MOs, SSp-bfd, SSs, Au. Area AM was injected twice (S3 Fig). The parcellation (names and locations of the areas) is based on Wang et al. [70] (S2 Fig) but differs from those used in Fig 4D and associated analyses.

### Histology

Four days after the tracer injection, mice were deeply anesthetized with an overdose of Ketamine/Xylazine and perfused through the heart with phosphate buffered saline, followed by 1% paraformaldehyde (PFA) in 0.1 M phosphate buffer (PB, pH 7.4). Immediately after, the cortex was dissected from the rest of the brain, completely unfolded, flat-mounted and post fixed overnight in 4% PFA at 4°C. Next, the tissue was cryoprotected in 30% sucrose and cut at 40 μm on a freezing microtome in the tangential plane.

### Imaging and Neuronal Counts

To survey the injection site and the distribution of labeled neurons across cortical areas, sections were wet-mounted in PB and imaged in St. Louis under a dissection scope equipped for UV- and red-fluorescence illumination. For plotting DY labeled neurons, the sections were permanently mounted onto glass slides and stored at 4°C. The distribution of DY-labeled neurons was analyzed in Bron. Plots of DY neurons were made at 20× under a fluorescence microscope equipped for UV illumination (excitation: 387–398 nm, emission: 435–475 nm), using the Mercator software package running on ExploraNova technology. Labeled neurons were contained in 12–16 sections per hemisphere. Digital charts of the coordinates of DY labeled neurons across each section were stored in the computer. Next, the regional pattern in the density of PVtdT expression was imaged under fluorescence optics. Finally, the sections were stained for Nissl substance, imaged under bright field illumination and superimposed onto the digital maps of DY and PVtdT fluorescence. In Bron, all the images were acquired using MorphoStrider software (ExploraNova).

### Alignments and Segmentation

The digital charts were saved in PDF files and were scaled in Adobe Illustrator. The charts and the corresponding images were brought to a common scale, allowing reconstruction of the sections. Sections were stacked in order, and then aligned. The landmark for the alignment of the sections was the injection site, followed by rotation around this point, allowing a 3-D reconstruction of the flattened brain. The injected area was delimited, as were the borders of the neocortex.

### Automated Processing

Automated processing was performed using in-house software, written in Python. For each case, the positions of labeled neurons inside neocortex, but outside the injected area (i.e., extrinsic neurons) were extracted in digital format, The fraction of labeled neurons per area (FLN) was estimated as the number of labelled neurons extrinsic to the injected area expressed as a fraction of the total number of labeled neurons in the cortical hemisphere [39]. Unlike the template matching procedure used in previous studies [5,6] we parcellated each cortex individually based on multiple markers expressed across different tangential sections. In a stepwise procedure we first used density differences in the expression of PV-tdT- labeled cell bodies and processes to delineate borders of single areas such as V1, S1, S2, Au, PD, UF, PV, GU, ORBI, MM. RSD, MOp, MOs, and ENTm (S2 Fig). Next, the PVtdT-expression pattern was used to outline regions which from previous studies are known to include multiple areas. The list includes (1) LM, LI, P, POR, 36p; (2) AL, LLA, RL, A, AM, PM; (3) TEp, TEa, ECT, PERI; (4) AIp, AId, AIv; and (5) ACAd, ACAv, PL, ILA, ORBm, FRP (S2 Fig). Each of these regions was further partitioned into areas based on the topographical distribution of DY labeled neurons, the size and location relative to readily identifiable areas, the rhinal sulcus, the crest of the medial wall [70–74] and the cytoarchitecture revealed by Nissl staining [5,75,76].

### Adapted Segmentation on Flattened Brain

The segmentation shown in Fig 4D was carried out in Adobe Illustrator, combining Allen Brain Atlas boundary criteria (visualized with Brain Explorer 2) with photos of PVtdT and Nissl staining, for one section of the flattened mouse brain. The contours of the cortical areas are non-self-intersecting closed polygons; therefore, computing their centroids is straightforward. The distances between areas were considered as the distances between their respective centroids.

### Datasets

The Allen Brain Institute (ABI) dataset was collected on their website, offered as link in their original research publication [5]. The University of Southern California (USC) matrix was, on the other hand, extracted directly from their original article [6]. The ABI mouse atlas possesses 40 isocortical areas according to their Supplemental Table 1. Out of these 40 areas, 2 did not correspond to any line or column in the data as structured in S1 Fig (i.e. the connectivity matrix). An additional 4 areas were not considered as primary target of an injection by the authors, leading to our decision to exclude them from our analysis. We then extracted from the ABI data a 34 × 34 weighted and directed connectivity matrix.

The larger USC dataset has a finer grained parcellation than that of ABI, although based on the same fundamental scheme. We contracted the USC final matrix down to a level of 42 × 42 by merging areas together in both rows and columns so as to obtain a squared matrix.

At this point, the ABI and USC matrices had 33 areas in common, which corresponds to 97% and 79% of their full respective matrices. A similar parcellation scheme was extracted out of the two datasets, allowing complete, connection-by-connection comparison between the two matrices, see S1 Fig for the final connectivity matrix.

### Technical Considerations

The database in the mouse has been generated following tracer injections in all cortical areas. The macaque data, however, was obtained from 29 injections using a 91-area atlas. Because in macaque we are using an edge-complete subgraph, the statistical features are predicted to reflect those of the, as yet unavailable, fully connected graph. However, the presently available dataset cannot give complete information on detailed areal relationships, such as for example the full membership of the cortical core.

### Variance Comparisons of Distance Distributions

Fig 7C shows the histograms of connection distances for mouse and macaque after normalization by the mean distance for each species. By construction, both distributions have mean equal to 1 and can be reasonably well described by truncated normal distributions. When fitting the distributions by maximum likelihood using functions from the truncnorm package [77] in R [78], the variance of the macaque normalized distances appears smaller than for the mouse data with a ratio of 0.608. Is this significant? First, we examine this question with an F-test on the ratio of variances. The test is two-sided because we do not specify a priori which variance is greater. This is a more conservative approach. The F-statistic is the variance ratio with degrees of freedom (405, 527) giving a highly significant *p =* 1.58 × 10^−7^. The test assumes normality, however. To verify the conclusion, then, we performed a permutation test that does not make the normality assumption [79]. In short, we permute the macaque and mouse labels a large number of times and recomputed the variance ratio for each new permutation. Under the null hypothesis that both distance distributions are the same, we expect a large number of variance ratio estimates on the permuted datasets that are more extreme than the variance ratio computed on the data. The *p*-value is computed from the proportion of ratio estimates more extreme or equal to the obtained value. For ratios, the definition of more extreme is based on the values that are less than the estimate and greater than its reciprocal. We include the ratio estimate from the dataset in the distribution of permutation estimates. S7 Fig shows the value of the ratio of variances for 100,000 permutations of the two datasets. The vertical line indicates the value obtained from the data, which is lower than all of the other values of the permutation distribution, indicating that the obtained ratio is highly unlikely under the hypothesis that both distributions are the same. The *p*-value is indicated in the graph. The *p*-value is smaller than 10^−5^, which is the resolution of the test for 100,000 permutations. Thus, the width of the distribution of distances for macaque is significantly narrower than that for mouse.

### Probability of a Connection as a Function of Distance

To analyze the density of connectivity with distance, we estimate the probability of a connection with distance. This can be done with a logistic regression. By performing the analysis on the binary connectivity (that is, presence/absence of a connection) at each distance, no binning is involved. Standard logistic regression implemented via a Generalized Linear Model with a binomial family [80] specifies that the expected value of the connection probability is related to a linear predictor through a link function that is often taken to be the log of the odds ratio or logit function. The model fit would be

$$g\left(E\left(Y\;=\;1\right)\right)\;=\;\beta_{0,i}+\beta_{1,i}\text{Distance}$$

where *Y* is a binary variable indicating whether a connection is present between two areas, *g* is the link function, here log(*p*/(1 − *p*))with *p* the expected value or probability of a connection, and *β*_0,*i*_ and *β*_1,*i*_ are intercept and slope, respectively, of the linear predictor, with *i* varying with the species. There is no a priori reason to suppose, however, that the sigmoid function of distance that this model implies will provide an adequate description of the change in probability with distance. To allow for a more flexible description of this relation, we fit the data with a Generalized Additive Model (GAM) using a binomial family [81]. The GAM model is given by

$$g\left(E\left(Y\;=\;1\right)\right)\;=\;I_{\text{mouse}}f_{\text{mouse}}\left(\text{Distance}\right)+I_{\text{macaque}}f_{\text{macaque}}\left(\text{Distance}\right)$$

where *f*_*i*_ are smooth functions of the covariates constructed from sums of spline curves with increasing complexity and *I*_*i*_ are indicator variables taking on the value of 1 for *i* = mouse (or, respectively, macaque) and 0 otherwise. The complexity (or wiggliness) of the fitted model is controlled by including a penalty in the fitting criterion based on the integrated square of the second derivatives of the *f*’s. The choice of degree of penalization (or smoothness) is controlled by minimizing a criterion related to prediction error (i.e., fitting some of the data and calculating the error on the remaining portion) called the un-biased risk estimator (UBRE) that is closely related to Aikake’s Information Criterion (AIC). Like AIC, UBRE favors a model that maximizes the predictability of future rather than the actual data and serves to minimize the tendency to overfit the data. The fits were performed with functions from the mgcv package [81] in R [78]. The estimates of the smooth curves for macaque and mouse are plotted in Fig 9C and 9D for macaque and mouse, respectively, with twice the estimated standard errors of the fits. To estimate the significance of the differences between the two estimates, we also fit the simpler nested model in which a single smooth curve described connectivity dependence with distance for both species. A likelihood ratio test of the nested models gave a *χ*^2^(2.26) = 54.04 with *p* = 3.05 × 10^−12^, strongly supporting that the differences in the curves are significant. Note that the generalization of degrees of freedom in the case of GAM fits are not necessarily integer valued. We extended this analysis to consider the connection probability as a smooth function of both distance and similarity. The GAM framework is used again but now to model surfaces of two variables, here giving the log-odds ratio of the connection probabilities as function of similarity and normalized distance. The model is given as

$$g\left(E\left(Y\;=\;1\right)\right)\;=\;I_{\text{mouse}}f_{\text{mouse}}\left(\text{Distance},\text{Similarity}\right)+I_{\text{macaque}}f_{\text{macaque}}\left(\text{Distance},\text{Similarity}\right),$$

where *f*_*i*_ are now smooth 2D functions of the covariates constructed from sums of spline surfaces with increasing complexity and *I_i_*, as before, are indicator variables taking the value of 1 for *i* = mouse (or, respectively, macaque) and 0 otherwise.

Contour plots of the estimates of the connection probability as a function of normalized distance and similarity are shown in Fig 9A and 9B. The color gradient indicates connection probability, passing from high (yellow, near 1) to low (green, near 0) connection probability. The curves indicate estimates of contours of constant connection probability (notated on the curves as probability values).

To evaluate the significance of the species difference displayed in Fig 9A and 9B, we also fit the simpler nested model in which a single smooth surface described connectivity dependence with distance and similarity for both species. A likelihood ratio test of the nested models gave a *χ*^2^(8.4) = 61.6 with *p* = 3.5 × 10^−10^, strongly supporting that the difference in the surfaces are significant. Note that as above the generalization of degrees of freedom in the case of GAM fits are not necessarily integer valued.

### Connectivity Similarity Indices

The method used to compute binary similarity indices with macaque data has been described previously [4]. Our published macaque database is made of 29 injected areas for a 91 parcellation scheme, thus giving a 91 × 29 connectivity matrix. In this context, only the in-degrees of injected areas are completely known, the out-degrees of source areas remaining incomplete. Therefore, if one wants to compare macaque and mouse using a degree-based binary similarity measure, one has to restrict oneself to in-degrees, in order to use complete data. For this reason, we detail here only the in-degree based similarity measurement calculations. The union of ABI and USC databases used here provides information about all 33 areas in terms of the in and out-going connections between 33 areas. We compared the similarity of the input pattern of pairs of areas by evaluating the number of sources areas from which both receive projections or neither do (i.e., similarity implies both projections exist or are absent; dissimilarity implies one is absent and the other is present). We define a normalized in-link similarity measure, $S_{xy}^{\text{in}}$ as follows: For any pair of areas (*x*, *y*), let $n_{xy}^{\text{in}}$ denote the number of projecting areas from which either both *x* and *y* or neither *x* nor *y* receive an incoming link. Because $n_{xy}^{\text{in}}\leq 33$, we compute the ratio $n_{xy}^{\text{in}}/33$ for every area pair (*x*, *y*). Clearly, this number will depend on the in-degrees of *x* and *y*, denoted by $k_{x}^{\text{in}}$ and $k_{y}^{\text{in}}\;(\;0\leq k_{x\left(y\right)}^{\text{in}}\leq 33)$. We define the in-link similarity as:

$$S_{xy}^{\text{in}}\;=\;\frac{n_{xy}^{\text{in}}}{33}-p_{xy}^{\text{in}},$$

where $p_{xy}^{\text{in}}$ is the expected value of the ratio ($n_{xy}^{\text{in}}/33$) if the incoming connections of *x* and *y* were distributed uniformly at random across the 33 source areas. Thus:

$$p_{xy}^{in}\;=\;\left(\frac{k_{x}^{\text{in}}}{33}\right)\left(\frac{k_{y}^{\text{in}}}{33}\right)+\left(1-\frac{k_{x}^{\text{in}}}{33}\right)\left(1-\frac{k_{y}^{\text{in}}}{33}\right),$$

where the first term is the probability that both *x* and *y* receive a link from a given source, and the second term is the probability that neither of them receive a link from a given source.

## Supporting Information

S1 Fig Binary connectivity matrix of the mouse. The matrix here is the union between published data of [5] and [6] (see Materials and Methods).
(PDF)

S2 Fig Identification of injection sites. Retrogradely labelled neurons after injection of DY into V1 in flat-mounted cerebral cortex of PVtdT expressing transgenic mouse. **a)** Tangential section though layer 4 showing high density of PVtdT expression (white) in visual (V1) auditory (Au), barrel-(S1), dorsal retrosplenial-(RSD) and medial entorhinal cortex (ENTm). The yellow spot within the shoeprint-shaped V1 marks the DY injection site. The inset (**a’**) shows visual cortex at higher magnification. The boot-shaped intensely PVtdT expressing adjoining the lateral border of V1 contains areas LM, P, LI and POR. The sparsely PVtdT expressing belt adjoining the anterior border of LM and surrounding the rest of V1 contains areas AL, RL, A, AM and PM. **b)** Image of the same section as in (**a**) taken at a longer exposure time to show DY-labelled neurons (yellow spots) in extrastriate visual areas POR, P, LM, LI, AL, RL, A, PM and AM. Notice, that the cell clusters are localized to the lower peripheral quadrant of the visual field [71]. The position of each map relative to the PVtdT labelled surrounding areas was used to assign injection sites to specific visual areas. Abbreviations: A (anterior area), ACAd (dorsal anterior cingulate area), AL (anterolateral area), AM (anteromedial area), Au (auditory area), CLA (claustrum), ENTl (lateral entorhinal area), ENTm (medial entorhinal area), Hip (hippocampus), LI (laterointermediate area), LM (lateromedial area), MM (mediomedial area), MOp (primary motor cortex), MOs (secondary motor cortex), OB (olfactory bulb), OT (olfactory tubercle), P (posterior area), PIR (piriform cortex), PM (posteromedial area), POR (postrhinal area), RL (rostrolateral area), PM (posteromedial area), RSD (dorsal retrosplenial area), S1 (primary somatosensory area), S2 (secondary somatosensory area), V1 (primary visual cortex).
(JPG)

S3 Fig Locations of injected areas in the flattened mouse isocortex. (EPS)

S4 Fig In- and out-degree distributions. The degrees (both in- and out-) are ranked and arranged decreasingly in the plots. The macaque connectivity data was generated via retrograde tracing. In this case usually a single target is injected per animal, revealing all the incoming connections and thus the in-degree to the injected target. Accordingly, the in-degree sequence (green symbols and line, panel **a**) will show the variability of in-degrees between the individuals injected. The out-degree sequence, however, is a population sample, as the only way to find all targets (out-links) for a given source is to combine all the individual injections. This is the green line in panel **b**. Clearly, the out-degree sequence shows much less variability. In both panels, the red marks are coming from individual EDR networks, all with *λ*^mac^ = 0.19 mm^−1^. The black markers show the average in-degrees and out-degrees over the the EDR model network realizations (200 realizations). The mouse tracing data (panels **c** and **d**) is dominated by anterograde tracing, in which case the in-degrees are the population sample data (panel **c**), whereas the out-links show variability between the individual animals (panel **d**). The red marks and the black are for the corresponding EDR model (with the same description for colors as for the macaque) in the mouse with *λ*^mus^ = 0.78 mm^−1^ (200 realizations).
(PDF)

S5 FigMotif distributions compared to the EDR model as a null-model.Bars show $\text{ln}\frac{m_{\text{data}}}{m_{\text{model}}}$, where *m* denotes the count of each possible three-motif in the empirical connectome and its corresponding EDR model, respectively. Counts from the models are averaged over 1,000 trials, black lines represent 95% confidence intervals.(PDF)

S6 Fig Wire length minimization. The vertical line shows the total wire length in the empirical network (**data**). The histogram is constructed from the total wire lengths in a set of networks obtained by randomly permuting the areas of the empirical network (**rand**).
(EPS)

S7 Fig Permutation test of Macaque/Mouse distance variance ratio. Macaque and mouse have significantly different distributions. Black line, empirical data; number of permutations: 100,000.
(EPS)

S1 Text Glossary of graph theory terms used in the article. (DOCX)

## Abbreviations

CDR: constant distance rule
EDR: exponential distance rule
FLN: fraction of labeled neurons
GAM: Generalized Additive Model
IT: information technology
RMS: Root mean square
WM: white matter

## References

[1] Sporns O. Networks of the brain. Cambridge, Mass: MIT Press; 2011.

[2] Fregnac Y, Bathellier B. Cortical Correlates of Low-Level Perception: From Neural Circuits to Percepts. Neuron. 2015;88(1):110–26. 10.1016/j.neuron.2015.09.041 26447576

[3] Buckner RL, Krienen FM. The evolution of distributed association networks in the human brain. Trends Cogn Sci. 2013;17(12):648–65. 10.1016/j.tics.2013.09.017 24210963

[4] Ercsey-Ravasz M, Markov NT, Lamy C, Van Essen DC, Knoblauch K, Toroczkai Z, et al A predictive network model of cerebral cortical connectivity based on a distance rule. Neuron. 2013;80(1):184–97. 10.1016/j.neuron.2013.07.036 24094111 PMC3954498

[5] Oh SW, Harris JA, Ng L, Winslow B, Cain N, Mihalas S, et al A mesoscale connectome of the mouse brain. Nature. 2014;508(7495):207–14. 10.1038/nature13186 24695228 PMC5102064

[6] Zingg B, Hintiryan H, Gou L, Song MY, Bay M, Bienkowski MS, et al Neural networks of the mouse neocortex. Cell. 2014;156(5):1096–111. 10.1016/j.cell.2014.02.023 24581503 PMC4169118

[7] Northcutt RG, Kaas JH. The emergence and evolution of mammalian neocortex. Trends Neurosci. 1995;18(9):373–8. 7482801 10.1016/0166-2236(95)93932-n

[8] Zhang K, Sejnowski TJ. A universal scaling law between gray matter and white matter of cerebral cortex. Proc Natl Acad Sci U S A. 2000;97(10):5621–6. 10792049 10.1073/pnas.090504197 PMC25878

[9] Allman J. Evolving brains. New York: Freeman, W. H.; 2000.

[10] Murre JM, Sturdy DP. The connectivity of the brain: multi-level quantitative analysis. Biol Cybern. 1995;73(6):529–45. 8527499 10.1007/BF00199545

[11] Karbowski J. How does connectivity between cortical areas depend on brain size? Implications for efficient computation. J Comput Neurosci. 2003;15(3):347–56. 14618069 10.1023/a:1027467911225

[12] Karbowski J. Optimal wiring principle and plateaus in the degree of separation for cortical neurons. Phys Rev Lett. 2001;86(16):3674–7. 11328051 10.1103/PhysRevLett.86.3674

[13] Stevens CF. How Cortical Interconnectedness Varies with Network Size. Neural Computation. 1989;1(4):473–9.

[14] Ringo JL. Neuronal interconnection as a function of brain size. Brain Behav Evol. 1991;38(1):1–6. 1657274 10.1159/000114375

[15] Cherniak C. Component placement optimization in the brain. J Neurosci. 1994;14(4):2418–27. 8158278 10.1523/JNEUROSCI.14-04-02418.1994 PMC6577144

[16] Cherniak C. Neural wiring optimization. Prog Brain Res. 2012;195:361–71. 10.1016/B978-0-444-53860-4.00017-9 22230636

[17] Cherniak C, Changizi M, Won Kang D. Large-scale optimization of neuron arbors. Phys Rev E Stat Phys Plasmas Fluids Relat Interdiscip Topics. 1999;59(5 Pt B):6001–9. 11969583 10.1103/physreve.59.6001

[18] Cherniak C, Mokhtarzada Z, Rodriguez-Esteban R, Changizi K. Global optimization of cerebral cortex layout. Proc Natl Acad Sci U S A. 2004;101(4):1081–6. 14722353 10.1073/pnas.0305212101 PMC327154

[19] Chklovskii DB. Optimal sizes of dendritic and axonal arbors in a topographic projection. J Neurophysiol. 2000;83(4):2113–9. 10758121 10.1152/jn.2000.83.4.2113

[20] Chklovskii DB, Koulakov AA. Maps in the brain: what can we learn from them? Annu Rev Neurosci. 2004;27:369–92. 15217337 10.1146/annurev.neuro.27.070203.144226

[21] Chklovskii DB, Schikorski T, Stevens CF. Wiring optimization in cortical circuits. Neuron. 2002;34(3):341–7. 11988166 10.1016/s0896-6273(02)00679-7

[22] Klyachko VA, Stevens CF. Connectivity optimization and the positioning of cortical areas. Proc Natl Acad Sci U S A. 2003;100(13):7937–41. 12796510 10.1073/pnas.0932745100 PMC164691

[23] Koulakov AA, Chklovskii DB. Orientation preference patterns in mammalian visual cortex: a wire length minimization approach. Neuron. 2001;29(2):519–27. 11239440 10.1016/s0896-6273(01)00223-9

[24] Raj A, Chen YH. The wiring economy principle: connectivity determines anatomy in the human brain. PLoS ONE. 2011;6(9):e14832 10.1371/journal.pone.0014832 21915250 PMC3168442

[25] Rivera-Alba M, Vitaladevuni SN, Mishchenko Y, Lu Z, Takemura SY, Scheffer L, et al Wiring economy and volume exclusion determine neuronal placement in the Drosophila brain. Curr Biol. 2011;21(23):2000–5. 10.1016/j.cub.2011.10.022 22119527 PMC3244492

[26] Kaiser M, Hilgetag CC, van Ooyen A. A simple rule for axon outgrowth and synaptic competition generates realistic connection lengths and filling fractions. Cereb Cortex. 2009;19(12):3001–10. 10.1093/cercor/bhp071 19435708

[27] Ringo JL, Doty RW, Demeter S, Simard PY. Time is of the essence: a conjecture that hemispheric specialization arises from interhemispheric conduction delay. Cereb Cortex. 1994;4(4):331–43. 7950307 10.1093/cercor/4.4.331

[28] Herculano-Houzel S, Mota B, Wong P, Kaas JH. Connectivity-driven white matter scaling and folding in primate cerebral cortex. Proc Natl Acad Sci U S A. 2010;107(44):19008–13. 10.1073/pnas.1012590107 20956290 PMC2973896

[29] Wen Q, Chklovskii DB. Segregation of the brain into gray and white matter: a design minimizing conduction delays. PLoS Comput Biol. 2005;1(7):e78 16389299 10.1371/journal.pcbi.0010078 PMC1323466

[30] Markov NT, Ercsey-Ravasz MM, Ribeiro Gomes AR, Lamy C, Magrou L, Vezoli J, et al A weighted and directed interareal connectivity matrix for macaque cerebral cortex. Cereb Cortex. 2014;24(1):17–36. 10.1093/cercor/bhs270 23010748 PMC3862262

[31] Wang X-J, Kennedy H. Brain structure and dynamics across scales: in search of rules. Curr Opin Neurobiol. 2016;37:92–8. 10.1016/j.conb.2015.12.010 26868043 PMC5029120

[32] Kennedy H, Knoblauch K, Toroczkai Z. Why data coherence and quality is critical for understanding interareal cortical networks. Neuroimage. 2013;80:37–45. 10.1016/j.neuroimage.2013.04.031 23603347

[33] Song HF, Kennedy H, Wang XJ. Spatial embedding of structural similarity in the cerebral cortex. Proc Nat Acad Sci USA. 2014;111(46):16580–5. 10.1073/pnas.1414153111 25368200 PMC4246295

[34] Bressler SL. Inferential constraint sets in the organization of visual expectation. Neuroinformatics. 2004;2(2):227–38. 15319518 10.1385/NI:2:2:227

[35] Passingham RE, Stephan KE, Kotter R. The anatomical basis of functional localization in the cortex. Nat Rev Neurosci. 2002;3(8):606–16. 12154362 10.1038/nrn893

[36] Felleman DJ, Van Essen DC. Distributed hierarchical processing in the primate cerebral cortex. Cereb Cortex. 1991;1(1):1–47. 1822724 10.1093/cercor/1.1.1-a

[37] Markov NT, Ercsey-Ravasz MM, Gariel MA, Dehay C, Knoblauch A, Toroczkai Z, et al The tribal networks of the cerebral cortex In: Chalupa LM, Berardi N, Caleo M, Galli-Resta L, Pizzorusso T, editors. Cerebral Plasticity. Cambridge MA: MIT Press; 2011 p. 275–90.

[38] Milo R, Shen-Orr S, Itzkovitz S, Kashtan N, Chklovskii D, Alon U. Network motifs: simple building blocks of complex networks. Science. 2002;298(5594):824–7. 12399590 10.1126/science.298.5594.824

[39] Markov NT, Misery P, Falchier A, Lamy C, Vezoli J, Quilodran R, et al Weight Consistency Specifies Regularities of Macaque Cortical Networks. Cereb Cortex. 2011;21(6):1254–72. 10.1093/cercor/bhq201 21045004 PMC3097985

[40] Markov NT, Ercsey-Ravasz M, Van Essen DC, Knoblauch K, Toroczkai Z, Kennedy H. Cortical high-density counter-stream architectures. Science. 2013;342(6158):1238406 10.1126/science.1238406 24179228 PMC3905047

[41] Roberts JA, Perry A, Lord AR, Roberts G, Mitchell PB, Smith RE, et al The contribution of geometry to the human connectome. Neuroimage. 2016;124(Pt A):379–93. 10.1016/j.neuroimage.2015.09.009 26364864

[42] Knoblauch K, Ercsey-Ravasz M, Kennedy H, Toroczkai Z. The brain in space In: Kennedy H, Van Essen D, Christen Y, editors. Micro-, meso- and macro- connectomics of the brain. 22: Springer, Heidelberg; 2014. p. In press. 28590672

[43] Zeki S. The Ferrier Lecture 1995 behind the seen: the functional specialization of the brain in space and time. Philos Trans R Soc Lond B Biol Sci. 2005;360(1458):1145–83. 16147515 10.1098/rstb.2005.1666 PMC1609195

[44] Dehaene S, Changeux JP. Experimental and theoretical approaches to conscious processing. Neuron. 2011;70(2):200–27. 10.1016/j.neuron.2011.03.018 21521609

[45] Erdős PL, Miklόs I, Toroczkai Z. A simple Havel-Hakimi type algorithm to realize graphical degree sequences of directed graphs. Electronic J Combinatorics. 2010;17(1):R66.

[46] Artzy-Randrup Y, Fleishman SJ, Ben-Tal N, Stone L. Comment on "Network motifs: simple building blocks of complex networks" and "Superfamilies of evolved and designed networks". Science. 2004;305(5687):1107; author reply 10.1126/science.1099334 15326338

[47] Markov NT, Ercsey-Ravasz M, Lamy C, Ribeiro Gomes AR, Magrou L, Misery P, et al The role of long-range connections on the specificity of the macaque interareal cortical network. Proc Natl Acad Sci U S A. 2013;110(13):5187–92. 10.1073/pnas.1218972110 23479610 PMC3612613

[48] Kaas J. Why is brain size so important: design problems and solutions as neocortex gets bigger or smaller. Brain and Mind. 2000;1:7–23.

[49] Jacob F. Evolution and tinkering. Science. 1977;196(4295):1161–6. 860134 10.1126/science.860134

[50] Kaas JH. The Evolution of Brains from Early Mammals to Humans. Wiley Interdiscip Rev Cogn Sci. 2013;4(1):33–45. 23529256 10.1002/wcs.1206 PMC3606080

[51] Geschwind DH, Rakic P. Cortical evolution: judge the brain by its cover. Neuron. 2013;80(3):633–47. 10.1016/j.neuron.2013.10.045 24183016 PMC3922239

[52] Striedter GF. Principles of brain evolution. Sunderland, MA: Sinauer Associates; 2005.

[53] Cahalane DJ, Charvet CJ, Finlay BL. Modeling local and cross-species neuron number variations in the cerebral cortex as arising from a common mechanism. Proc Natl Acad Sci U S A. 2014;111(49):17642–7. 10.1073/pnas.1409271111 25422426 PMC4267349

[54] Charvet CJ, Cahalane DJ, Finlay BL. Systematic, cross-cortex variation in neuron numbers in rodents and primates. Cereb Cortex. 2015;25(1):147–60. 10.1093/cercor/bht214 23960207 PMC4259279

[55] Cahalane DJ, Clancy B, Kingsbury MA, Graf E, Sporns O, Finlay BL. Network structure implied by initial axon outgrowth in rodent cortex: empirical measurement and models. PLoS ONE. 2011;6(1):e16113 10.1371/journal.pone.0016113 21264302 PMC3019165

[56] Finlay BL, Uchiyama R. Developmental mechanisms channeling cortical evolution. Trends Neurosci. 2015;38(2):69–76. 10.1016/j.tins.2014.11.004 25497421

[57] Henriksen S, Pang R, Wronkiewicz M. A simple generative model of the mouse mesoscale connectome. Elife. 2016;5. 10.7554/eLife.12366 PMC4807721 26978793

[58] Henderson JA, Robinson PA. Geometric effects on complex network structure in the cortex. Phys Rev Lett. 2011;107(1):018102 21797575 10.1103/PhysRevLett.107.018102

[59] Song S, Sjostrom PJ, Reigl M, Nelson S, Chklovskii DB. Highly nonrandom features of synaptic connectivity in local cortical circuits. PLoS Biol. 2005;3(3):e68 15737062 10.1371/journal.pbio.0030068 PMC1054880

[60] Feller W. An Introduction to Probability Theory and Its Applications. second ed: John Wiley & Sons; 1971.

[61] Park SY, Bera AK. Maximum entropy autoregressive conditional heteroskedasticity model. J Econometrics. 2009;150:219–30.

[62] Chung KL. Elementary Probability Theory. New York: Springer-Verlag; 2010.

[63] Reveley C, Seth AK, Pierpaoli C, Silva AC, Yu D, Saunders RC, et al Superficial white matter fiber systems impede detection of long-range cortical connections in diffusion MR tractography. Proc Natl Acad Sci U S A. 2015;112(21):E2820–8. 10.1073/pnas.1418198112 25964365 PMC4450402

[64] Donahue CJ, Sotiropoulos S, Jbabdi S, Herandez-Ferandez M, Behrens T, Kennedy H, et al Using diffusion tractography to predict cortical connection strength and distance: a quantitative comparison with tracers in the monkey. J Neurosci. 2016;in press. 10.1523/JNEUROSCI.0493-16.2016 PMC4916250 27335406

[65] Delbeuck X, Collette F, Van der Linden M. Is Alzheimer's disease a disconnection syndrome? Evidence from a crossmodal audio-visual illusory experiment. Neuropsychologia. 2007;45(14):3315–23. 17765932 10.1016/j.neuropsychologia.2007.05.001

[66] Friston KJ, Frith CD. Schizophrenia: a disconnection syndrome? Clin Neurosci. 1995;3(2):89–97. 7583624

[67] Bullmore E, Sporns O. The economy of brain network organization. Nat Rev Neurosci. 2012;13(5):336–49. 10.1038/nrn3214 22498897

[68] Hippenmeyer S, Vrieseling E, Sigrist M, Portmann T, Laengle C, Ladle DR, et al A developmental switch in the response of DRG neurons to ETS transcription factor signaling. PLoS Biol. 2005;3(5):e159 15836427 10.1371/journal.pbio.0030159 PMC1084331

[69] Madisen L, Zwingman TA, Sunkin SM, Oh SW, Zariwala HA, Gu H, et al A robust and high-throughput Cre reporting and characterization system for the whole mouse brain. Nat Neurosci. 2010;13(1):133–40. 10.1038/nn.2467 20023653 PMC2840225

[70] Wang Q, Sporns O, Burkhalter A. Network analysis of corticocortical connections reveals ventral and dorsal processing streams in mouse visual cortex. J Neurosci. 2012;32(13):4386–99. 10.1523/JNEUROSCI.6063-11.2012 22457489 PMC3328193

[71] Wang Q, Burkhalter A. Area map of mouse visual cortex. J Comp Neurol. 2007;502(3):339–57. 17366604 10.1002/cne.21286

[72] Wang Q, Gao E, Burkhalter A. Gateways of ventral and dorsal streams in mouse visual cortex. J Neurosci. 2011;31(5):1905–18. 10.1523/JNEUROSCI.3488-10.2011 21289200 PMC3040111

[73] Garrett ME, Nauhaus I, Marshel JH, Callaway EM. Topography and areal organization of mouse visual cortex. J Neurosci. 2014;34(37):12587–600. 10.1523/JNEUROSCI.1124-14.2014 25209296 PMC4160785

[74] Issa JB, Haeffele BD, Agarwal A, Bergles DE, Young ED, Yue DT. Multiscale optical Ca2+ imaging of tonal organization in mouse auditory cortex. Neuron. 2014;83(4):944–59. 10.1016/j.neuron.2014.07.009 25088366 PMC4242551

[75] Franklin KBJ, Paxinos G. The Mouse Brain in Stereotaxic Coordinates. 3rd ed San Diego: Academic Press; 2007.

[76] Van De Werd HJ, Rajkowska G, Evers P, Uylings HB. Cytoarchitectonic and chemoarchitectonic characterization of the prefrontal cortical areas in the mouse. Brain Struct Funct. 2010;214(4):339–53. 10.1007/s00429-010-0247-z 20221886 PMC2862954

[77] Trautmann H, Steuer D, Mersmann O, Bornkamp B. truncnorm: Truncated normal distribution. R package version 1.0–7. http://cran.r-/project.org/package=truncnorm. 2014.

[78] R Development Core Team. R: A language and environment for statistical computing. Vienna, Austria: R Foundation for statistical computing http://www.r-project.org/ 2015.

[79] Efron B, Tibshirani RJ. An introduction to the bootstrap. New York: Chapman and Hall; 1993.

[80] McCullagh P, Nelder JA. Generalized linear models. 2nd ed Boca Raton: Chapman & Hall/CRC; 1989.

[81] Wood SN. Generalized Additive Models: An Introduction with R. Hall Ca, editor: CRC Press; 2006.

