# Supplementary figures and images for: Spatial Embedding and Wiring Cost Constrain the Functional Layout of the Cortical Network of Rodents and Primates
Source: PLoS Biol. 2016 Jul 21;14(7):e1002512. doi: 10.1371/journal.pbio.1002512 (PMC4956175; doi:10.1371/journal.pbio.1002512)

TO

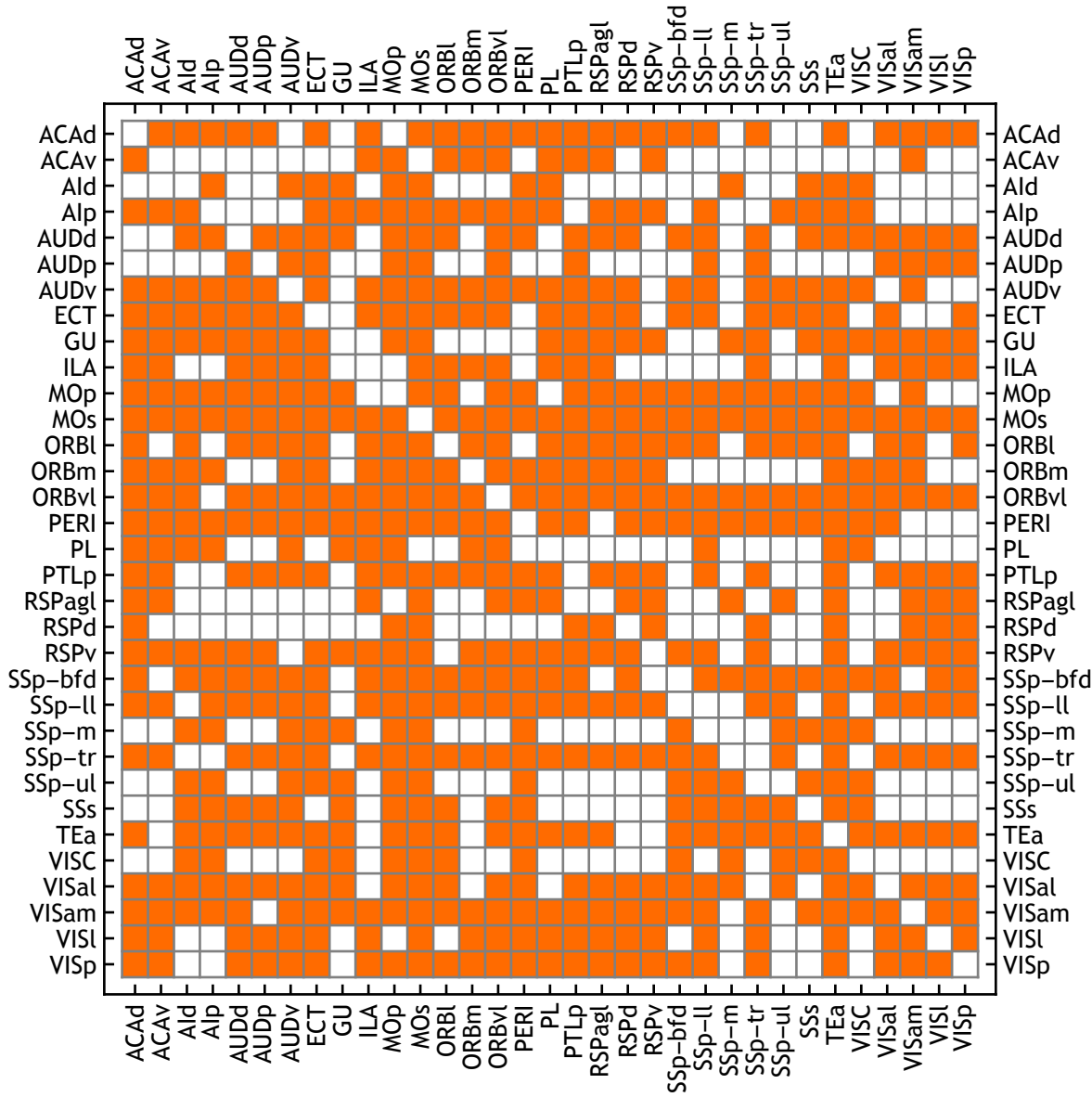

Supplement: S1 Fig — The matrix here is the union between published data of [5] and [6] (see Materials and Methods). (PDF) [file pbio.1002512.s001.pdf]

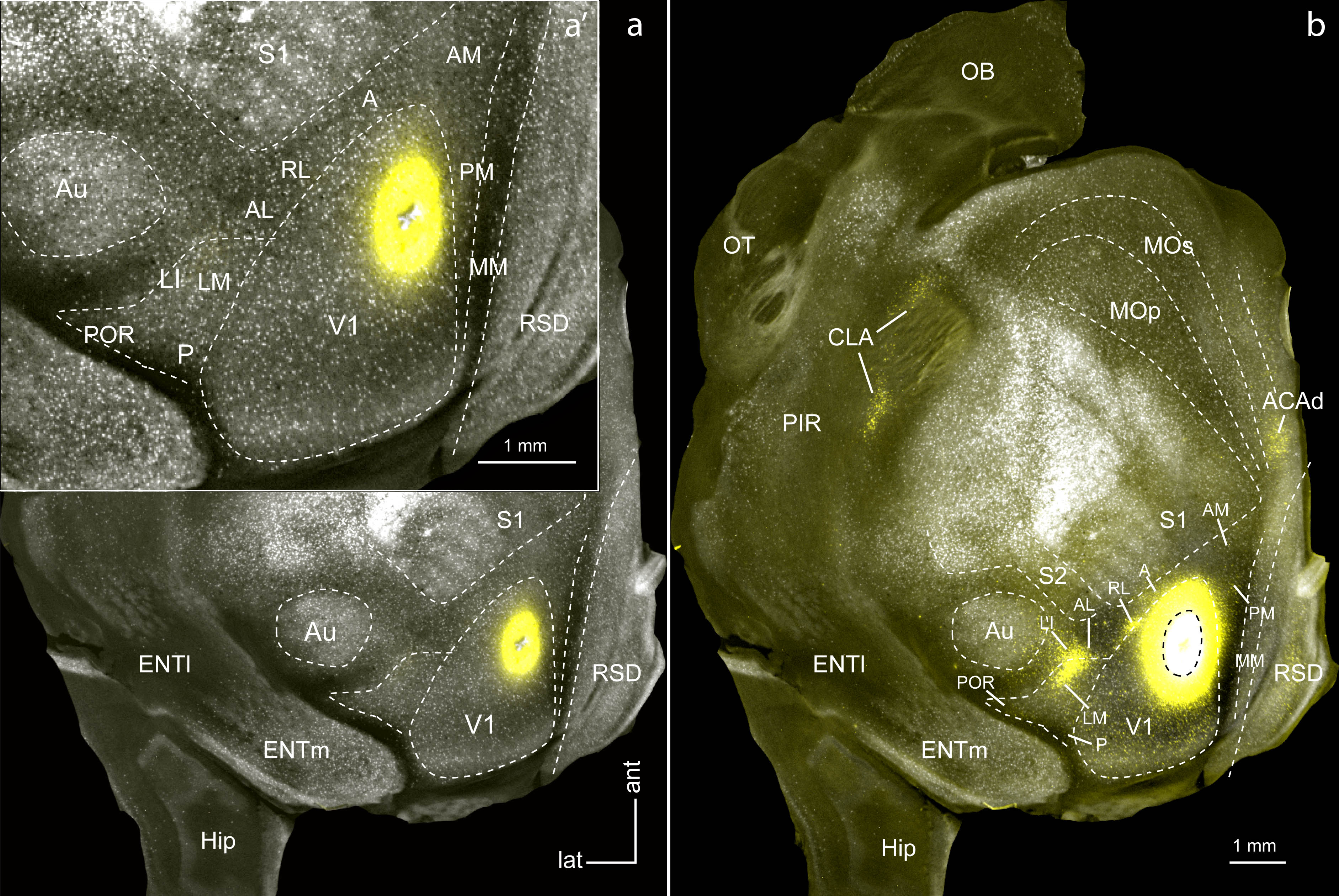

Supplement: S2 Fig — Retrogradely labelled neurons after injection of DY into V1 in flat-mounted cerebral cortex of PVtdT expressing transgenic mouse. a) Tangential section though layer 4 showing high density of PVtdT expression (white) in visual (V1) auditory (Au), barrel-(S1), dorsal retrosplenial-(RSD) and medial entorhinal cortex (ENTm). The yellow spot within the shoeprint-shaped V1 marks the DY injection site. The inset (a’) shows visual cortex at higher magnification. The boot-shaped intensely PVtdT expressing adjoining the lateral border of V1 contains areas LM, P, LI and POR. The sparsely PVtdT expressing belt adjoining the anterior border of LM and surrounding the rest of V1 contains areas AL, RL, A, AM and PM. b) Image of the same section as in (a) taken at a longer exposure time to show DY-labelled neurons (yellow spots) in extrastriate visual areas POR, P, LM, LI, AL, RL, A, PM and AM. Notice, that the cell clusters are localized to the lower peripheral quadrant of the visual field [71]. The position of each map relative to the PVtdT labelled surrounding areas was used to assign injection sites to specific visual areas. Abbreviations: A (anterior area), ACAd (dorsal anterior cingulate area), AL (anterolateral area), AM (anteromedial area), Au (auditory area), CLA (claustrum), ENTl (lateral entorhinal area), ENTm (medial entorhinal area), Hip (hippocampus), LI (laterointermediate area), LM (lateromedial area), MM (mediomedial area), MOp (primary motor cortex), MOs (secondary motor cortex), OB (olfactory bulb), OT (olfactory tubercle), P (posterior area), PIR (piriform cortex), PM (posteromedial area), POR (postrhinal area), RL (rostrolateral area), PM (posteromedial area), RSD (dorsal retrosplenial area), S1 (primary somatosensory area), S2 (secondary somatosensory area), V1 (primary visual cortex). (JPG) [file pbio.1002512.s002.jpg]

macaque

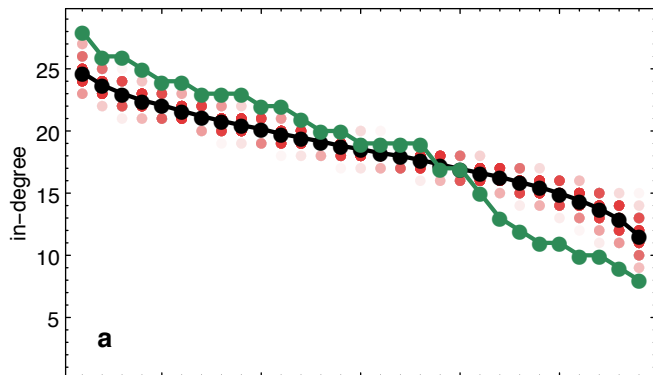

mouse

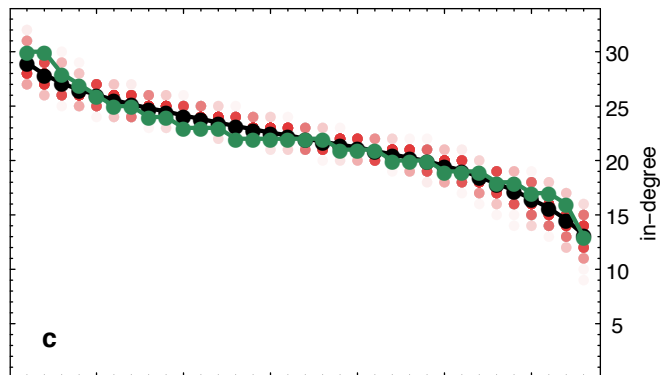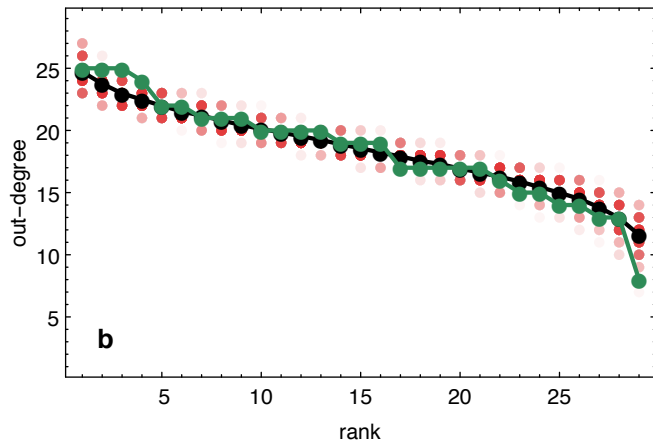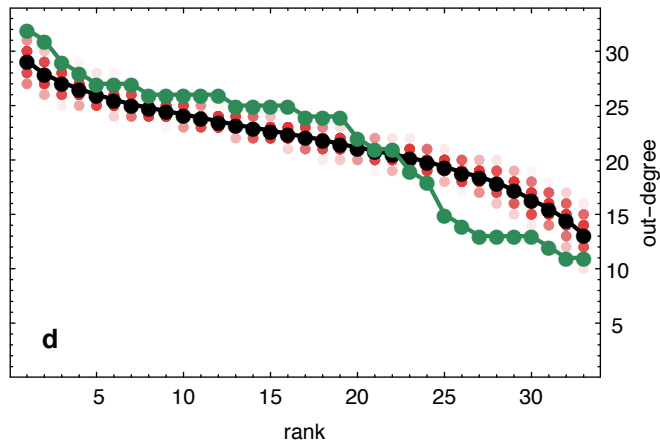

Supplement: S4 Fig — The degrees (both in- and out-) are ranked and arranged decreasingly in the plots. The macaque connectivity data was generated via retrograde tracing. In this case usually a single target is injected per animal, revealing all the incoming connections and thus the in-degree to the injected target. Accordingly, the in-degree sequence (green symbols and line, panel a) will show the variability of in-degrees between the individuals injected. The out-degree sequence, however, is a population sample, as the only way to find all targets (out-links) for a given source is to combine all the individual injections. This is the green line in panel b. Clearly, the out-degree sequence shows much less variability. In both panels, the red marks are coming from individual EDR networks, all with λmac = 0.19 mm−1. The black markers show the average in-degrees and out-degrees over the the EDR model network realizations (200 realizations). The mouse tracing data (panels c and d) is dominated by anterograde tracing, in which case the in-degrees are the population sample data (panel c), whereas the out-links show variability between the individual animals (panel d). The red marks and the black are for the corresponding EDR model (with the same description for colors as for the macaque) in the mouse with λmus = 0.78 mm−1 (200 realizations). (PDF) [file pbio.1002512.s004.pdf]

motifs:  $\ln \frac{\text{data}}{\text{model}}$

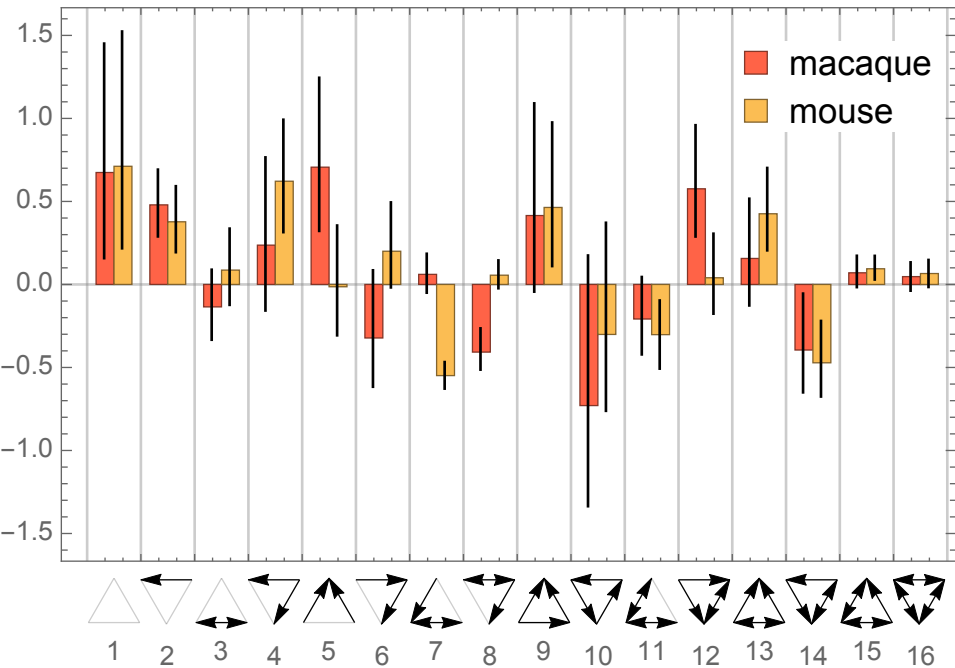

Supplement: S5 Fig — Bars show lnmdatammodel, where m denotes the count of each possible three-motif in the empirical connectome and its corresponding EDR model, respectively. Counts from the models are averaged over 1,000 trials, black lines represent 95% confidence intervals. (PDF) [file pbio.1002512.s005.pdf]
