# Supplementary material for: Spatial Embedding and Wiring Cost Constrain the Functional Layout of the Cortical Network of Rodents and Primates
Source: PLoS Biol. 2016 Jul 21;14(7):e1002512. doi: 10.1371/journal.pbio.1002512 (PMC4956175; doi:10.1371/journal.pbio.1002512)
Supplement: S1 Text — (DOCX) [file pbio.1002512.s008.docx]

**Glossary**

**Complete graph** is a graph (see graph) in which the maximum possible number of edges exist. An undirected complete graph on $N$ nodes has $N(N-1)/2$ edges, whereas a directed graph $N(N-1)$ edges. The density (see density) of a complete graph is always 1.

**Cliques** are complete subgraphs of a network. In other words, a clique is a set of nodes which have the maximum possible number of connections between them. For example, a 3-clique in an undirected graph is simply a triangle, and in a directed graph it is a triangle with two, oppositely oriented edges along each one of its sides (6 directed edges in total). Cliques, especially the large ones, are crucial components of a network as they identify maximally interconnected regions, usually with functional significance.

**Density** of a graph or network is given by the fraction between the number of its edges and the maximum number of edges the graph could support. For example, in a directed graph on $N$ nodes, the maximum number of edges it could support is $N(N-1)$, because every node can connect to $N-1$ other nodes, and there are $N$ of them in total. Thus its density is $\rho=\frac{M}{N\left( N-1 \right)}$, where $M$ is the number of edges.

**EDR**, the *exponential distance rule*, refers to the experimental observation that the number of neuronal projections of length $d$ found in a cortical hemisphere behaves as $e^{-\lambda d}$ . It is not to be confused with the distance dependence of the interareal connection strengths, the latter measured as fraction of labelled neurons, FLNs (see FLN).

**FLN** refers to the *fraction of labelled neurons* found in a given cortical area: $\mathrm{FLN}=k_{\mathrm{area}}/k_{\mathrm{total}}$ where $k_{\mathrm{area}}$ is the number of labelled neurons in the area of interest and $k_{\mathrm{total}}$ is the total number of labelled neurons, excluding those in the injected area. FLN values are used as a measure of interareal connection strengths.

**Graphs** are an abstract mathematical description of connections between objects (called *nodes* or *vertices*). For example, specifying the connections (or their lack) for every brain area pair, one defines a graph. Graphs can be represented graphically as stick-and-disks figures (nodes are disks, sticks are edges), or given by the so-called connectivity (adjacency) matrices, which is simply a two-dimensional array of 0s (no connection) and 1s (connection) between all pairs of nodes.

$\boldsymbol{k}$**-motifs** are the directed connectivity patterns that $k$ nodes can have. There are three 2-motifs because two nodes can be unconnected, unidirectionally connected or bidirectionally connected. There are 16 different 3-motifs, shown in Fig. 4a. Motif analysis studies how frequently different motifs appear in a directed network, often in comparison with a null model network.
